# Cardiac Pacemaker Cells Generate Cardiomyocytes from Fibroblasts in Long-Term Cultures

**DOI:** 10.1038/s41598-019-51001-6

**Published:** 2019-10-23

**Authors:** Shigeki Kiuchi, Akino Usami, Tae Shimoyama, Fuminori Otsuka, Sachiko Yamaguchi, Tomonori Nakamura, Shigeto Suzuki, Kageyoshi Ono

**Affiliations:** 1 Laboratory of Molecular Physiology and Pharmacology, Faculty of Pharma-Sciences, Teikyo University, Itabashi-Ku, Tokyo, 173-8605 Japan; 2 Laboratory of Molecular Environmental Health, Faculty of Pharma-Sciences, Teikyo University, Itabashi-Ku, Tokyo, 173-8605 Japan; 3 Department of Molecular Pharmacology and Pharmacotherapeutics, Graduate School of Pharmaceutical Sciences, Chiba University, Chuo-ku, Chiba, 260-8675 Japan; 4 Present Address: Sodegaura Satsuki-dai Hospital, Sodegaura, Chiba, 299-0246, Japan; 5 Present Address: Division of Pharmaceutical Care Sciences, Center for Social Pharmacy and Pharmaceutical Care Sciences, Faculty of Pharmacy, Keio University, Tokyo, 105-8512, Japan

**Keywords:** Differentiation, Cardiovascular biology

## Abstract

Because cardiomyocyte generation is limited, the turnover of cardiomyocytes in adult heart tissues is much debated. We report here that cardiac pacemaker cells can generate cardiomyocytes from fibroblasts *in vitro*. Sinoatrial node cells (SANCs) were isolated from adult guinea pig hearts and were cultured at relatively low cell densities. Within a week, a number of fibroblast-like cells were observed to gather around SANCs, and these formed spontaneously beating clusters with cardiomyocyte structures. The clusters expressed genes and proteins that are characteristic of atrial cardiomyocytes. Pharmacological blocking of pacemaker currents inhibited generation of action potentials, and the spontaneous beating were ceased by physically destroying a few central cells. Inhibition of beating during culture also hampered the cluster formation. Moreover, purified guinea pig cardiac fibroblasts (GCFs) expressed cardiac-specific proteins in co-culture with SANCs or in SANC-preconditioned culture medium under electrical stimulation. These results indicate that SANCs can generate cardiomyocytes from cardiac fibroblasts through the influence of humoral factor(s) and electrophysiological activities followed by intracellular Ca^2+^ oscillations. This potential of SANCs to generate cardiomyocytes indicates a novel mechanism by which cardiomyocytes turns over in the vicinity of pacemaker cells and could be exploited in the development of strategies for cardiac regenerative therapy in adult hearts.

## Introduction

To date, cardiomyocytes are known to be produced even in adult animals under certain *in vivo* physiological or pathological conditions, but the adult heart has a very limited capacity for regeneration^1^. In 25-year-old humans, cardiomyocytes are renewed at a rate of about 1% per year, and in 75-year-old humans, this rate is only 0.45%. Accordingly, 45% of cardiomyocytes are regenerated after birth, by the age of 50 or later^2^. Hence, although the heart can renew itself even after birth, the rate of renewal is insufficient to overcome massive losses of cardiomyocytes in cases of cardiac failure. To address this, the roles of various cardiac progenitor cells have been intensively investigated as potential sources for cardiogenesis during the lifetimes of humans^3–8^.

Loss or dysfunction of sinoatrial node cells (SANCs) leads to sick sinus syndrome or sinus node dysfunction, and these conditions are prevalent in the elderly. SANCs are present in limited areas and in limited numbers, with about 1,000 cells in guinea pigs^9^, 2,000 cells in cats^10^, 5,000 cells in rabbits^11^ and probably not more than 10,000 cells in humans^12^. Although intrinsic renewal of SANCs may also occur during one’s life, it remains unknown whether these limited numbers of SANCs remain alive and active without replacement throughout the human lifespan.

Apart from intrinsic renewal of cardiomyocytes, cardiogenesis from cardiac fibroblast cells^13,14^ has been proposed in studies of cardiac regenerative therapies^15^. Production of functional cardiomyocytes has been achieved following reprogramming of fibroblasts by gene transfer^14^ and exogenous chemical treatments^13,16^. These manipulations targeted at upregulating the expression of cardiac transcription factors and downstream genes, triggering the transcription of mRNAs that contribute to cardiomyocyte differentiation^17–19^. Among involved factors, epidermal growth factor (EGF) and vascular epithelial growth factor (VEGF) enhanced cardiomyocyte generation by activating intracellular pathways including Akt^20^.

In our initial examination of the behaviour of SANCs in culture, we found that spontaneously beating clusters of cardiomyocyte-like cells formed around SANCs that were obtained from adult guinea pig hearts and cultured at relatively low cell densities. These clusters had shapes distinct from re-aggregating neonatal myocytes that start to beat spontaneously in high cell-density culture^21^. In the present study, we analysed the characteristics of nascent cells in these clusters, identified their origins and investigated mechanisms by which SANCs create cardiomyocytes.

## Results

### Generation of beating cell clusters *in vitro*

**Figure 1:**
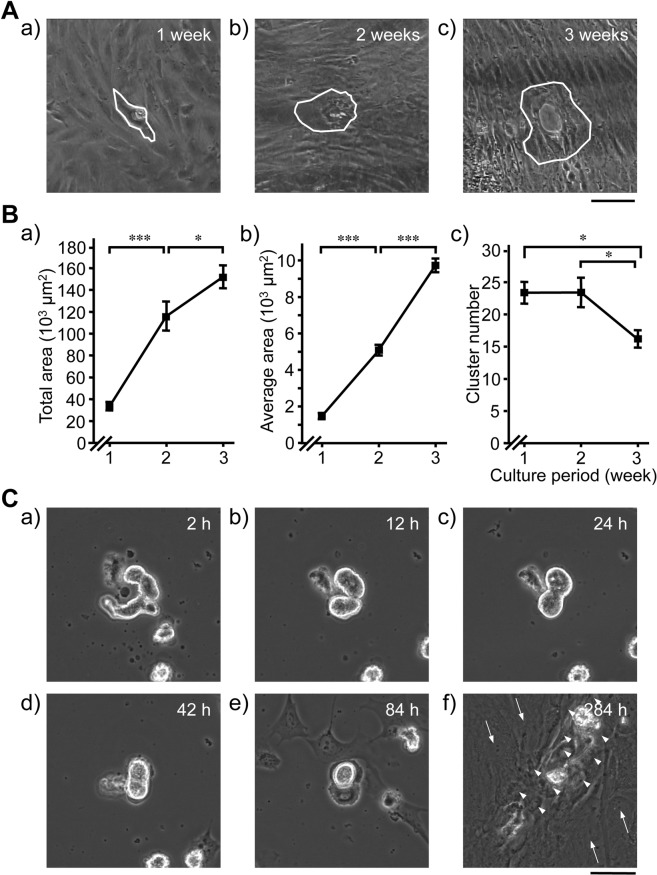
Emergence of synchronised and spontaneously beating cell clusters and their expansion during culture of adult sinoatrial node cells (SANCs). (**A**) Typical phase-contrast images of a SANC and its surrounding area after culture for 1 (a), 2 (b) and 3 weeks (c); a synchronised cell cluster with spontaneous beating was formed around the SANC by 1 week of culture (a). Continued culture resulted in gradual expansion of cell cluster sizes (b,c). Spontaneously beating clusters are outlined with white; *bar*, 100 µm. (**B**) Time course of the growth of beating cell clusters; a) total beating areas in the culture dish, (b) average areas of the clusters and (c) numbers of clusters in the culture dish; values are expressed as the mean ± standard errors of the mean (S.E.M.; n = 25). Vertical bars denote S.E.M.; Tukey’s test; *P < 0.05, ***P < 0.001. (**C**) Typical cell images were taken from time-lapse video recordings of morphological changes in SANCs and their surrounding areas during culture for 2–284 h. (a) Spontaneous beating of SANCs was maintained despite partial shrinking at 2 h after starting the culture. (b–e) Fusing of 2 adjacent SANCs was observed occasionally between 12 and 84 h in culture. Migration of fibroblast-like cells started at around 84 h. (f) A spontaneously beating cell cluster had formed and had markedly expanded by 284 h of culture. Beating cells (arrow heads) in cell clusters exhibited distinct morphology compared with neighbouring fibroblast-like flat cells that were outside the beating cluster (thin arrows); *bar*, 50 µm.

SANC suspensions contained SANCs and morphologically distinct atrial myocytes at almost equal numbers^22^. When SANC suspensions were cultured at a density of 1–2 × 10^4^ cells/cm^2^, 20–50 beating cells per cm^2^ were observed initially, and these continued to beat spontaneously for about 24 h. Most atrial myocytes did not attach to culture dishes and were thus removed upon replacement of the medium. A number of fibroblast-like flat polygonal cells were then observed around SANCs after 3 days, and these gradually formed clusters around the SANCs by 7 days (Fig. 1A-a, Supplementary Movie S1). Some of the clusters started beating spontaneously, and beating areas gradually expanded for up to 3 weeks (Fig. 1A-b,c,B-a, Supplementary Movie S2).

The beating clusters expanded with time (Fig. 1B-b), whereas their numbers decreased (Fig. 1B-c), indicating combining of the clusters. In addition, beating rates were similar to basal heart rates of adult guinea pigs, averaging 80–150 beats/min (bpm). Similar results were achieved in cultures of SANCs from guinea pigs that were older than 52 weeks old (body weight, 800–1,000 g). In contrast, such beating clusters were not formed around atrial myocytes; the few remaining atrial cells were shrunken and silent in the culture medium. Similarly, no beating clusters were formed when suspensions of left atrial cells were cultured; only fibroblast-like quiescent cells grew to confluence.

In time-lapse microscope experiments (Supplementary Movie S3), SANCs kept beating after shrinking a little from 2 to 24 h (Fig. 1C-a–c) in culture. Fibroblast-like cells then moved towards SANCs, and some attached to SANCs to form clusters by 84 h (Fig. 1C-e). Occasionally, pairs of adjoining SANCs appeared to fuse with each other during the period between 12 and 84 h (Fig. 1C-b–e). These clusters gradually expanded by 284 h and started beating (Fig. 1C-f) around approximately half of the originally seeded SANCs.

### Characterisation of spontaneously beating cell clusters

**Figure 2:**
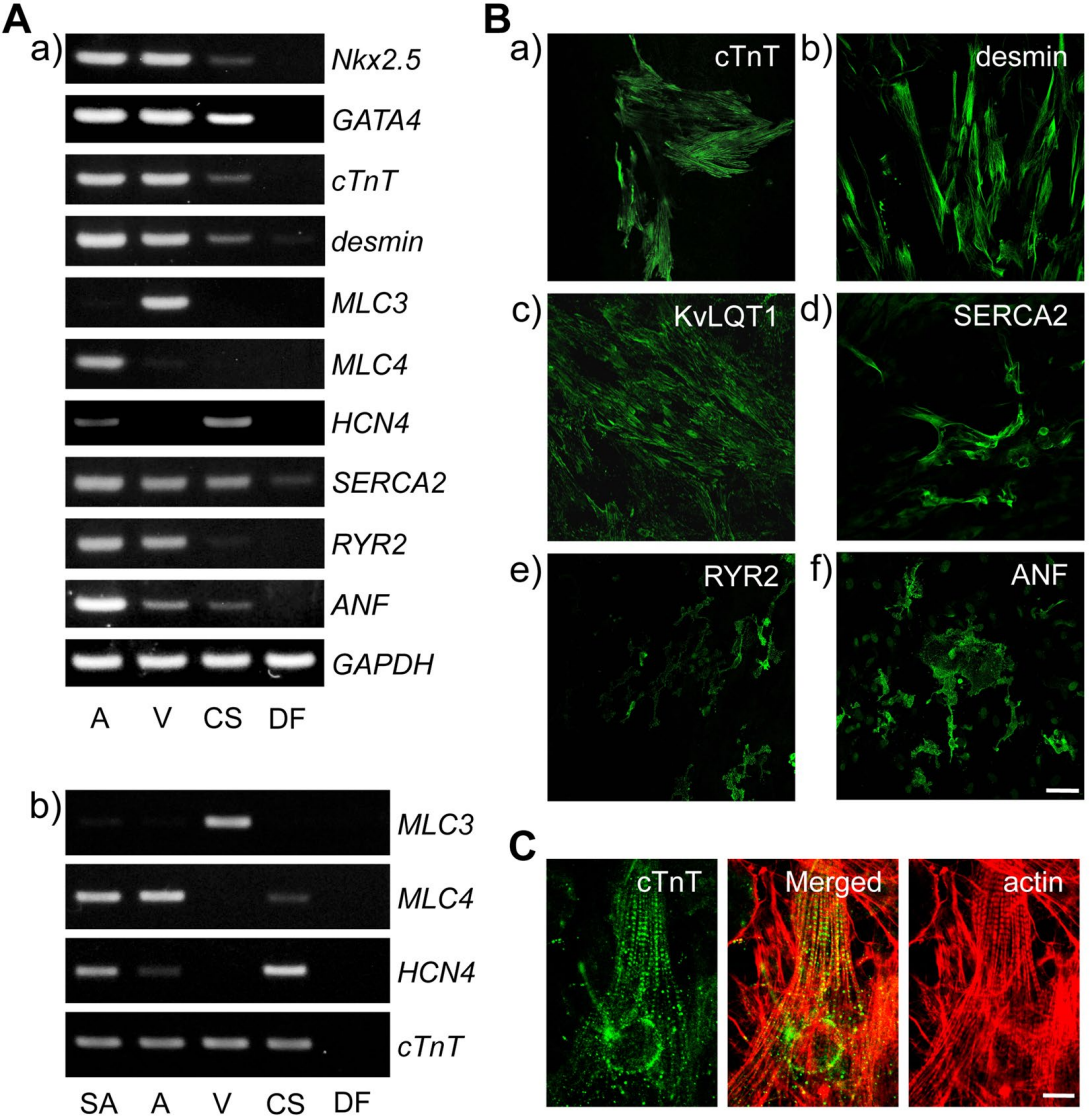
Characterisation of synchronised and spontaneously beating cell clusters formed during SANC culture. (**A**) Reverse transcription-polymerase chain reaction (RT-PCR) analysis of cardiac gene expression in cell clusters after culturing SANCs for 3 weeks; *Nkx2*.*5*, *GATA4*, *cTnT*, *desmin*, *MLC4*, *HCN4*, *SERCA2*, *RYR2* and *ANF* transcripts, but not those of *MLC3*, were detected in beating cell clusters during the culture of SANCs (lane ‘CS’). To compare expression levels of cardiac genes in cell clusters, *GAPDH* and *cTnT* were used as internal controls in panels a and b, respectively. SA, sinoatrial node tissue; A, atrial cell suspension; V, ventricular cell suspension; CS, cultured SANCs; DF, dermal fibroblasts. Full-length gels from which the images were cropped are given in Supplementary Figs S7–S9. (**B**) Immunocytochemical detection of cardiac proteins at 2 weeks of culture; cell clusters that had grown around SANCs expressed cTnT (a), desmin (b), KvLQT1 (c), SERCA2 (d), RYR2 (e) and ANF (f). (**C**) Fine striated sarcomeric patterns of cTnT (green) and actin (red) were observed after 3 weeks of culture; *bars*, 50 µm (**B**); 10 µm (**C**).

To characterize the cell clusters that had grown and started beating during the SANC culture, we determined the expression levels of cardiomyocyte-specific and/or cardiomyocyte-essential genes using reverse transcription-polymerase chain reaction (RT-PCR) analyses. The newly generated clusters expressed mRNAs for the cardiac transcription factors *Nkx2*.*5* and *GATA4*, the contractile and cytoskeletal proteins cardiac troponin T (*cTnT*) and desmin, the pacemaker channel hyperpolarisation-activated, cyclic nucleotide-gated cation channel 4 (*HCN4*) and the Ca^2+^ handling proteins sarco(endo)plasmic reticulum Ca^2+^-ATPase 2 (*SERCA2*) and ryanodine receptor 2 (*RYR2*), and atrial natriuretic factor (*ANF*), after 3 weeks of culture (Fig. 2A-a). Using *cTnT* as an internal standard, we observed abundant expression of myosin light chain 4 (*MLC4*) but not myosin light chain 3 (*MLC3*), indicating that atrial but not ventricular type cardiomyocytes were generated (Fig. 2A-b).

Consistent with the mRNA expression data, a wide range of cardiac proteins, including cTnT, desmin, voltage-dependent K^+^ channel α subunit (KvLQT1), SERCA2, RyR2 and ANF, were detected in immunocytochemical analyses as early as 1 week from the start of the culture (Supplementary Fig. S1). Areas that were positive for these proteins were subsequently enlarged over 2 weeks in culture (Fig. 2B-a–f). Moreover, some clusters that expressed these proteins grew further and exhibited fine striated sarcomeric constructs of cTnT and actin by 3 weeks culture (Fig. 2C). No cardiac proteins were detected in morphologically distinct fibroblast-like flat and thin cells outside the rims of the beating areas. Yet culture of isolated left atrial myocytes resulted in confluent growth of fibroblast-like cells that did not beat or express cardiac-related proteins. These observations suggested that SANCs, but not atrial myocytes, generate cardiomyocytes of nodal or atrial types.

### Properties of nascent beating cardiomyocyte clusters

**Figure 3:**
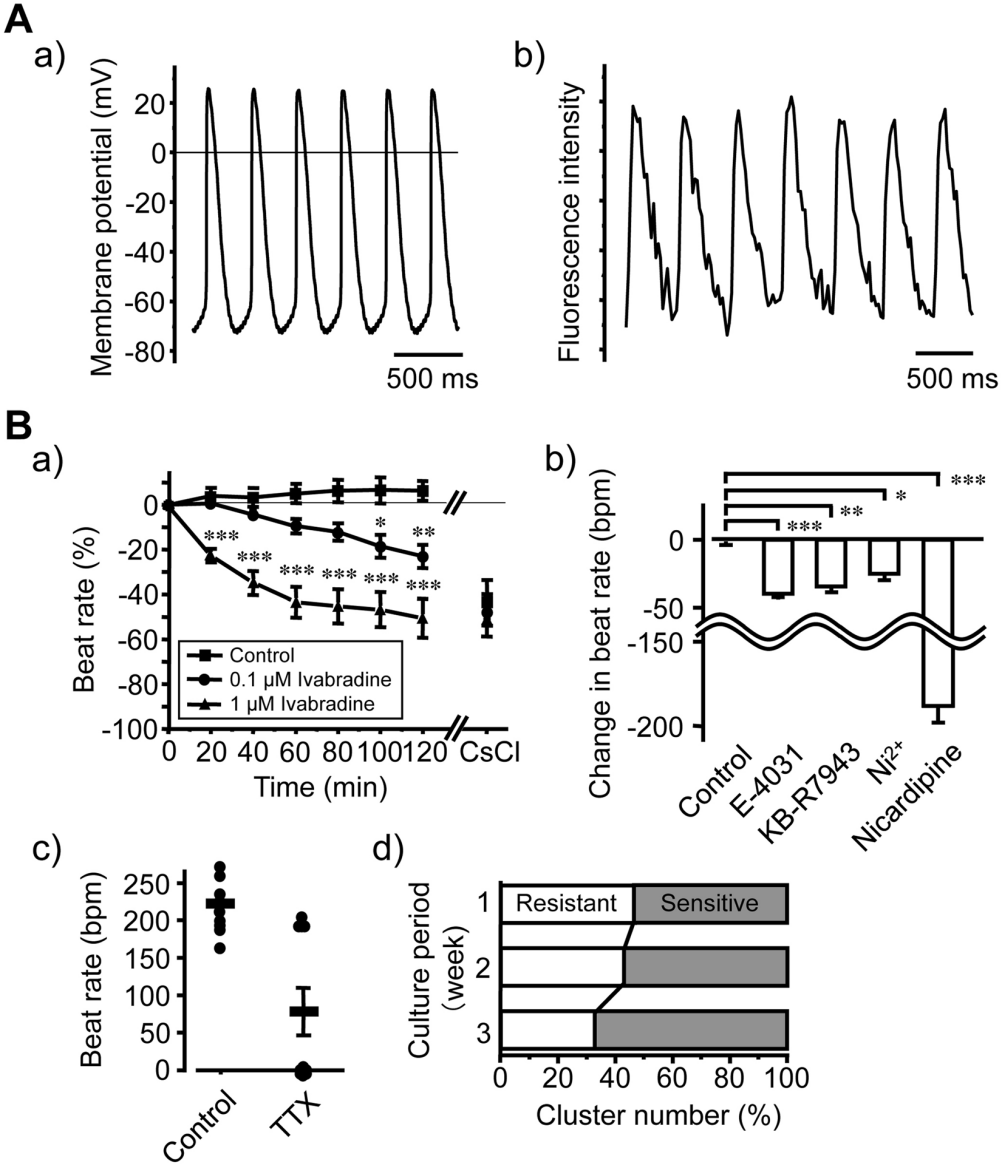
Electrophysiological and pharmacological characterisation of cardiomyocyte clusters in SANC cultures. (**A**) Action potentials were recorded from the centre of a spontaneously beating cardiomyocyte cluster after 3 weeks in culture using a whole-cell patch pipette (a). Intracellular Ca^2+^ transient from other clusters was recorded optically using Fluo-4 after 3 weeks of culture (b). (**B**) Analysis of ion channels and an exchanger during spontaneous beating of cardiomyocyte clusters in SANC cultures after 3 weeks; a) the *I*_f_ inhibitor ivabradine (0.1 µM,●; 1 µM, ▲) decreased beat rates gradually (n = 8) in a concentration-dependent manner, and addition of the ionic *I*_f_ inhibitor CsCl (3 mM) reduced beating rates completely. (b) The effects of the *I*_Kr_, *I*_Na/Ca_, *I*_CaT_ and *I*_CaL_ inhibitors E-4031 (3 µM), KB-R7943 (5 µM), Ni^2+^ (50 µM) and nicardipine (10 µM), respectively, on rates of spontaneous beating (n = 10); (c) Effects of tetrodotoxin (10 µM) on beating rates; horizontal lines indicate averages, and ticks indicate S.E.M. Tetrodotoxin-sensitive and tetrodotoxin-resistant clusters were present (n = 10). (d) Time-dependent increases in the proportions of tetrodotoxin-sensitive and tetrodotoxin-resistant cell clusters (n > 150) (chi-square test, *P* = 0.0413); data are expressed as mean ± S.E.M. Vertical bars denote S.E.M.; Dunnett’s test, *P < 0.05, **P < 0.01, ***P < 0.001, compared with control.

Beating cardiomyocyte clusters spontaneously generated action potentials, which were recorded from their central areas. They fired following the smooth transition after slow diastolic depolarisation (pacemaker potential). The action potential configuration was almost identical to that of ‘central SANCs’ (Fig. 3A-a)^22^. Electrical recordings from the periphery of cell clusters were largely unsuccessful because the cells were too thin to establish whole-cell patch-clamp recordings. Nonetheless, optical recordings of intracellular free Ca^2+^ concentrations revealed that intracellular Ca^2+^ transient was generated in a synchronised manner in many clusters, indicating functional synchronism (Fig. 3A-b, Supplementary Movie S4, S5). Physical destruction of single cells that appeared as drivers of the spontaneous excitation using the patch pipette tip slowed the beating of the whole cluster. The beating was completely abolished after crushing 1 or 2 more cells (Supplementary Fig. S2, Movie S6). These observations suggest that several clusters combined to synchronise with each other and that the combined cluster contained more than one pacemaker cells that drove the beating in the surrounding quiescent cardiomyocytes.

The inhibition of *I*_f_ using ivabradine (0.1 and 1 µM) significantly decreased beating rates of cardiomyocyte clusters in a concentration-dependent manner (Fig. 3B-a). Similarly, the *I*_Kr_ blocker E-4031 (3 µM), the *I*_Na/Ca_ inhibitor KB-R7943 (5 µM), the *I*_CaT_ blocker Ni^2+^ (50 µM) and the *I*_CaL_ blocker nicardipine (10 µM) significantly reduced spontaneous beating rates (Fig. 3B-b). Tetrodotoxin (10 µM) stopped spontaneous beating in a population of clusters but failed to do so in others, suggesting the presence of tetrodotoxin-sensitive and tetrodotoxin-resistant clusters (Fig. 3B-c). Moreover, the proportions of the tetrodotoxin-sensitive clusters increased over time in culture (Fig. 3B-d), as observed during the development of cardiomyocytes around hatching^23^.

Beating rate of the nascent cardiomyocyte clusters were found to be modulated by isoprenaline and acetylcholine as in physiological SANCs (Supplementary Fig. S3, Movie S7). The clusters also responded to endogenous biological peptides, such as endothelin-1 and calcitonin gene-related peptide, quite similar to their response to sinoatrial nodes in physiological conditions (Supplementary Fig. S4).

### A step for acquiring cardiac phenotypes in GCFs

**Figure 4:**
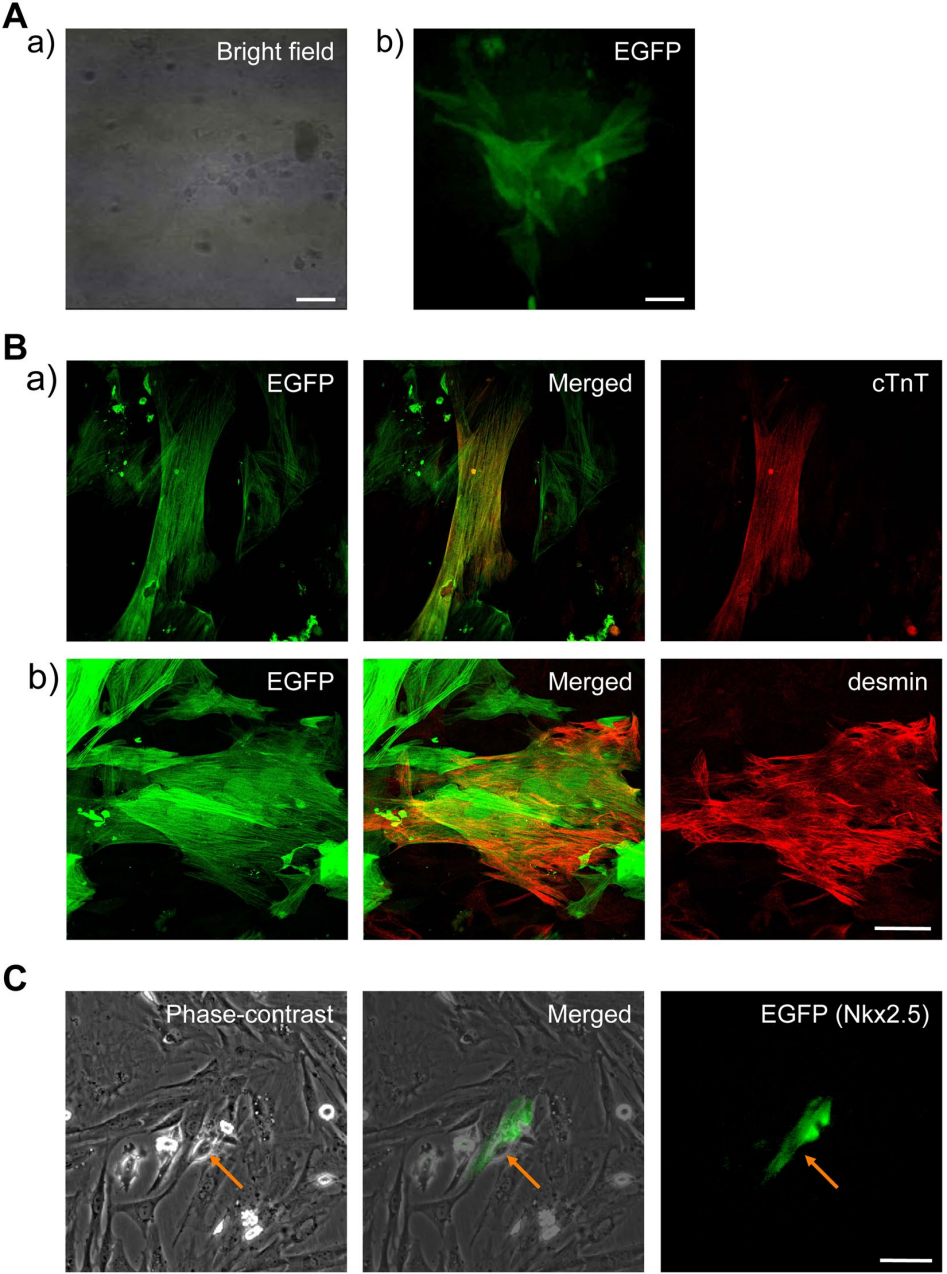
Acquisition of cardiac phenotypes in GCFs after co-culture with SANCs. (**A**) GCFs stably expressing enhanced green fluorescent protein (EGFP) started beating spontaneously after 2 weeks co-culture with SANCs. Typical bright-field (a) and fluorescence (b) images of GCFs are shown. (**B**) Expression of cardiac marker proteins in GCFs pre-labelled with EGFP; immunofluorescence images of cTnT (a, red) or desmin (b, red) in EGFP-labelled (a,b, green) GCFs. An image for the negative control for this experiment, i.e., in the absence of co-culture with SANCs, is presented vide infra. (**C**) Typical GCFs with Nkx2.5 expression, as indicated by the EGFP reporter signal in the vicinity of a SANC; pEGFP/Nkx2.5BD transfected GCFs were co-cultured with SANCs for 48 h. The arrow indicates one of SANCs that were originally placed in cultures. *bars*, 50 µm.

To determine whether nascent cardiomyocytes originated from cardiac fibroblasts, we cultured purified and EGFP-labelled GCFs with isolated SANCs. After 2 weeks in co-culture, several clusters of EGFP-labelled GCFs began to beat spontaneously (Fig. 4A). They expressed cTnT and desmin (Fig. 4B). When purified GCFs, which were prepared to express the *EGFP* reporter for Nkx2.5, were co-cultured with SANCs, some of these began to show Nkx2.5 signals in close proximity with SANCs at 48 h (Fig. 4C).

### Roles of intra- and extracellular signals

**Figure 5:**
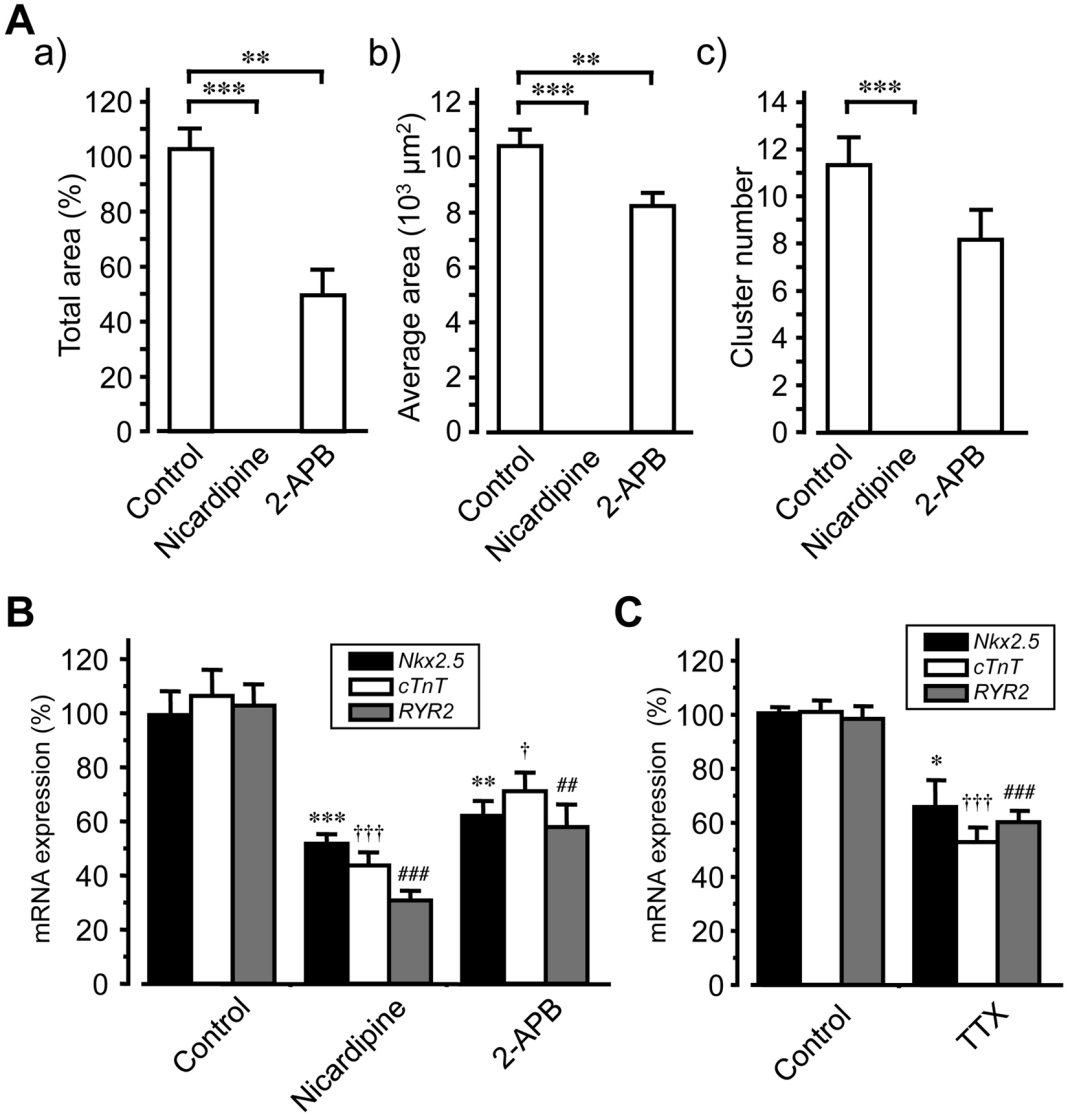
Suppression of cardiomyocyte generation by inhibiting entry or intracellular release of Ca^2+^. (**A**) Effects of nicardipine (10 µM) and 2-APB (3 µM) on the growth of spontaneously beating cardiomyocyte clusters after 3 weeks culture; total areas (panel a), average areas (panel b) and numbers (panel c) of spontaneously beating clusters (mean ± S.E.M., n = 6) are illustrated. B, C, effects of nicardipine (10 µM) and 2-APB (3 µM; panel B) and tetrodotoxin (10 µM, panel C) on *Nkx2*.*5*, *cTnT* and *RYR2* mRNA expression after 2 weeks of SANC culture (n = 6), as estimated using quantitative RT-PCR. Bars denote mean ± S.E.M. (n = 6); *^,†^*P* < 0.05; **^,##^*P* < 0.01; ^***,†††,###^*P* < 0.001. Symbols denote significant differences from corresponding control values (Dunnett’s test).

In further experiments, we manipulated intra- and extracellular signals and determined the effects on cardiomyocyte generation during SANC culture. Blockade of Ca^2+^ entry using nicardipine (10 µM) during 3 weeks culture of SANC suspensions totally suppressed the formation of beating clusters. Moreover, no cells were observed beating spontaneously around SANCs, even after removing nicardipine containing media and washing the cells. Similarly, the growth of cardiomyocyte clusters was significantly inhibited when Ca^2+^ release from the sarcoplasmic reticulum was blocked using 2-APB (3 µM; Fig. 5A-a–c). These observations were in agreement with suppressed *Nkx2*.*5*, *cTnT* and *RYR2* mRNA expression levels, which were significantly reduced already at 2 weeks in culture with nicardipine or 2-APB (Fig. 5B). Treatment of SANC cultures with tetrodotoxin for 2 weeks also significantly reduced the expression of *Nkx2*.*5*, *cTnT* and *RYR2* mRNA (Fig. 5C).

**Figure 6:**
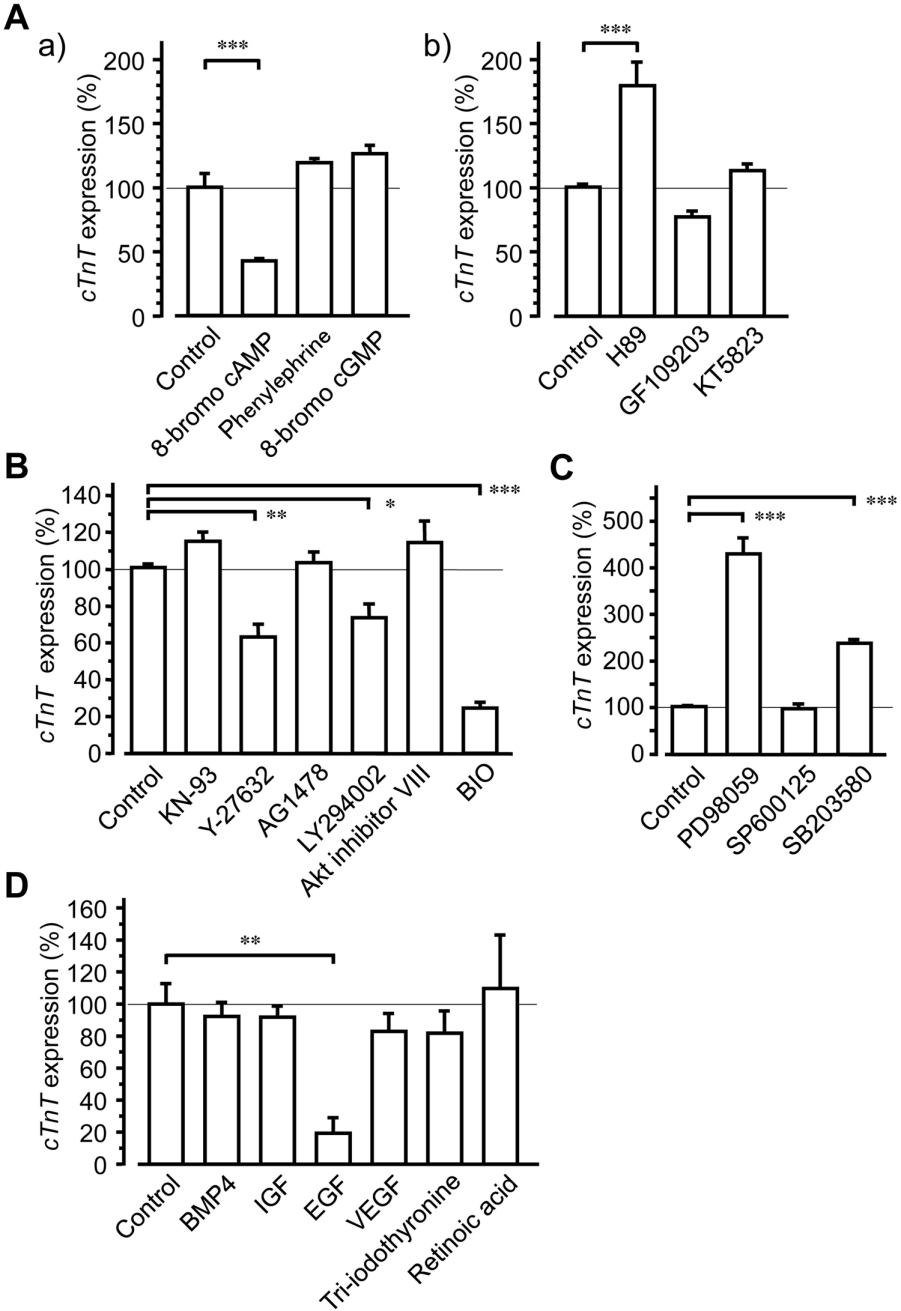
Roles of intracellular kinases and extracellular stimuli in *cTnT* expression in SANC cultures. The effects of various chemicals on *cTnT* expression levels in cell clusters were examined using quantitative RT-PCR after 3 weeks culture. (**A**) Activation and inhibition of protein kinases A, C and G; the effects of 8-bromo cAMP (500 µM), phenylephrine (3 µM) and 8-bromo cGMP (500 µM; n = 3) on *cTnT* expression levels are shown in panel a, and those of H89 (10 µM), GF109203 (1 µM) and KT5823 (400 nM; n = 6) are shown in panel b. (**B**) The roles of CaMK II, ROCK, EGFRK, PI_3_K, Akt and GSK3 were examined by analysing the effects of the corresponding inhibitors KN-93 (2 µM), Y-27632 (10 µM), AG1478 (1 µM), LY294002 (10 µM), Akt inhibitor VIII (1 µM) and BIO (3 µM), respectively, (n = 6). (**C**) Roles of ERK1/2, JNK and p38 were examined by analysing the effects of the inhibitors PD98059 (10 µM), SP600125 (10 µM) and SB203580 (10 µM), respectively, (n = 6). (**D**) The effects of BMP4 (10 ng/mL), IGF (10 ng/mL), EGF (100 ng/mL), VEGF (50 ng/mL), triiodothyronine (30 nM) and retinoic acid (1 µM; n = 3). Bars denote mean ± S.E.M.; Dunnett’s test; *P < 0.05, **P < 0.01, ***P < 0.001.

To identify intracellular signalling pathways that are involved in cardiomyocyte generation, we determined mRNA expression levels of the cardiomyocyte marker *cTnT*. Over 3 weeks culture, activation of protein kinase A (PKA) by 8-bromo cAMP (500 µM) significantly decreased *cTnT* expression, whereas activation of PKC or protein kinase G (PKG) using phenylephrine (3 µM) or 8-bromo cGMP (500 µM), respectively, failed to affect *cTnT* expression (Fig. 6A-a). Conversely, whereas inhibition of PKA by H89 (10 µM) increased *cTnT* expression significantly (Fig. 6A-b), inhibition of PKC or PKG using GF109203 (1 µM) or KT5823 (400 nM), respectively, did not.

Inhibition of ROCK, PI_3_K or GSK3 following treatments with Y-27632 (10 µM), LY294002 (10 µM) or BIO (3 µM), respectively, decreased *cTnT* expression significantly, whereas inhibition of CaMK II, EGF receptor kinase or Akt kinase by KN-93 (2 µM), AG1478 (1 µM) or Akt inhibitor VIII (1 µM), respectively, did not affect *cTnT* expression during SANC culture (Fig. 6B). In contrast, inhibition of ERK1/2 or p38 by PD98059 (10 µM) or SB203580 (10 µM), respectively, significantly increased *cTnT* expression, whereas inhibition of JNK by SP600125 (10 µM) had no effect (Fig. 6C). Finally, EGF treatments (100 ng/mL) decreased apparent *cTnT* expression during SANC culture, whereas treatments with BMP4 (10 ng/mL), IGF (10 ng/mL), VEGF (50 ng/mL), triiodothyronine (30 nM) and retinoic acid (1 µM) did not affect *cTnT* expression (Fig. 6D).

### Differentiation of GCFs into cardiomyocytes

**Figure 7:**
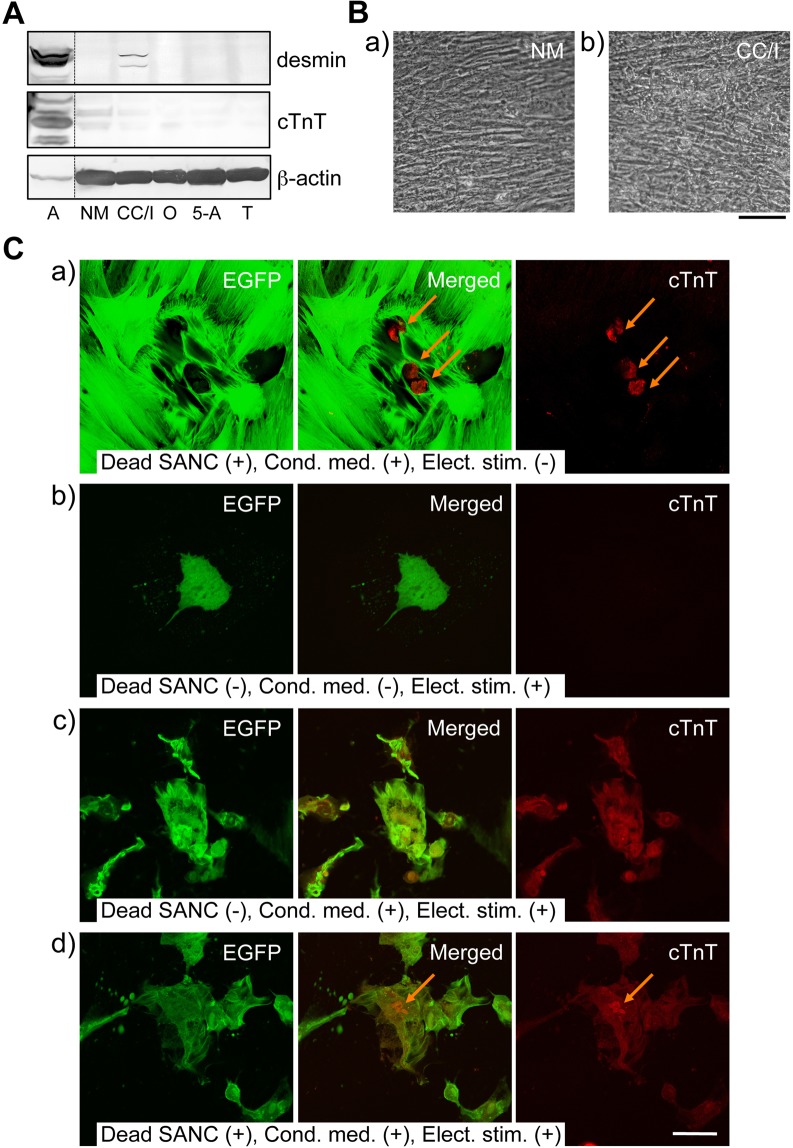
Changes in the properties of purified GCFs in various culture environments. (**A**) Expression of desmin in GCFs co-cultured with SANCs without cell–cell contact (CC/I); desmin was not detected when GCFs were monocultured without SANCs in normal medium (NM); cTnT expression was not detected under either condition. Oxytocin (100 nM, O), 5-azacytidine (10 µM, 5-A) and trichostatin A (10 pg/mL, T) failed to induce desmin expression in monocultured GCFs even after 3 weeks culture. A, atrium. Each lane contained a total of 50 µg of protein. Full-length blots are given in Supplementary Fig. S10. (**B**) Typical phase-contrast images of GCFs cultured in normal medium (NM) (a) or co-cultured with SANCs in the same dish, but with physical isolation by cellulose membranes with 1-µm pores (co-culture with isolation, CC/I) (b) for 3 weeks; C, Immunocytochemical detection of cTnT protein (red) in EGFP-labelled (green) GCFs cultured under various conditions for 1 week; a) co-cultures with pre-fixed SANCs in SANC-conditioned medium; cTnT was detected only in pre-fixed SANCs (arrows). (b,c) Culture with repetitive electrical field stimulation in normal (b) and SANC-conditioned (c) medium; (d) the same condition as in (c) but with pre-fixed SANCs (arrow). *bars*, 50 µm.

When purified GCFs and SANCs were co-cultured in chambers that were separated by cellulose membranes to prevent their direct contact, GCFs expressed desmin after 3 weeks (Fig. 7A, CC/I), although the overall morphology of cell clusters did not differ from those in GCF-alone mono cultures (Fig. 7B-a,b). In normal medium without the SANC-conditioning, GCFs failed to express desmin (Fig. 7A, NM). Moreover, neither oxytocin (100 nM), 5-azacytidine (10 µM) nor trichostatin A (10 pg/mL) induced the expression of desmin in these monocultured GCFs (Fig. 7A). Critically, none of these treatments or compounds induced the expression of cTnT protein, at a detectable level, or beating in GCFs.

When the purified and EGFP-labelled GCFs were co-cultured with formaldehyde-fixed SANCs for 1 week, they failed to express cTnT, even in the presence of SANC-conditioned medium (Fig. 7C-a). In contrast, although continuous electrical field stimulation of GCFs did not by itself induce cTnT expression in normal medium (Fig. 7C-b), it did induce cTnT expression in the SANC-conditioned medium (Fig. 7C-c,d).

## Discussion

In this study, we cultured SANCs isolated from adult guinea pigs and observed that a number of cells gathered around the SANCs, followed by construction of clusters that then started to beat synchronously. These clusters gradually expanded with culture time. Although cardiomyocytes isolated from neonatal hearts, but not from adult hearts, form spontaneously beating re-aggregates when cultured at relatively high densities^21^, the clusters found in our study were distinct from such three-dimensional re-aggregates of working cardiomyocytes. Beating clusters were generated around SANCs in culture, and this unique property was demonstrated even in SANCs from aged animals. Herein, the formation of contracting clusters was observed around SANCs, but not around atrial myocytes, indicating that the construction of beating cardiomyocyte clusters is not due to the re-aggregation of working cardiomyocytes originally put into the culture.

In our RT-PCR analyses of the beating cell clusters, the cardiomyocyte-specific mRNAs for *Nkx2*.*5*, *GATA4*, *cTnT*, *desmin*, *HCN4*, *SERCA2* and *RYR2* were all expressed. In addition, expression of the atrial-type mRNAs *MLC4* and *ANF* was observed, whereas the ventricular type *MLC3* mRNA^24^ was not expressed under these conditions. Correspondingly, in our immunocytochemical analyses, the beating clusters expressed various cardiac proteins, including cTnT, desmin, KvLQT1, SERCA2, RYR2 and ANF after only 1 week of culture. Collectively, these gene and protein expression profiles indicate that the beating clusters comprised nascent atrial-type cardiomyocytes with the SANCs that were probably present in the cultures initially. These proteins were re-localised after 2 weeks culture. Specifically, SERCA2 and RYR2 were initially detected in the cytoplasm but were found beneath the plasma membrane after 2 weeks culture. Subsequently, actin and cTnT were observed in fine striated sarcomeric structures, which emerged by 3 weeks of culture. These data suggest that nascent atrial-type cardiomyocytes translocate these proteins from as early as 1 week and then mature *in vitro*.

Beating rates of the present clusters were similar to basal heart rates of adult guinea pigs, and many clusters exhibited synchronised spikes in intracellular Ca^2+^ concentrations. In the present experiments, spontaneously generated action potentials with SANC-like configurations were recorded from single cells at the centres of clusters, and physical destruction of these central cells abolished beating of the whole cluster. These observations indicate that cells at the periphery of the clusters were electrically driven by a few pacemaker cells in the centre. The density of the clusters on the culture dishes was similar to that of SANCs remaining on the first day of culture, and immunocytochemical staining revealed expression of atrial cardiomyocyte proteins in most of the cells within the clusters. These findings suggest that limited numbers of SANC-type pacemaker cells, which are likely derived from original SANCs, are present in the centre of the clusters and are coupled electrically with the surrounding nascent atrial-type cardiomyocytes to construct syncytial cell clusters.

The inhibition of *I*_f_, *I*_Kr_, *I*_Na/Ca_, *I*_CaT_ or *I*_CaL_ significantly reduced spontaneous beating rates of clusters. Because these pharmacological treatments are known to inhibit pacemaker potentials and the generation of action potentials in SANCs in isolated intact hearts^25^, it was indicated that all corresponding ion channels are expressed functionally in the present cardiomyocyte clusters. Perhaps these drugs inhibited ion channels in the SANCs at the centres of clusters. Blocking of *I*_CaL_ may also have inhibited action potentials in nascent cardiomyocytes.

The beating was tetrodotoxin-sensitive in some clusters but was tetrodotoxin-resistant in others, and proportions of tetrodotoxin-sensitive clusters increased over 3 weeks culture. In a previous study, action potentials of cardiomyocytes were tetrodotoxin-resistant at the embryonic stage but became tetrodotoxin-sensitive with maturation of cardiomyocytes after hatching^23^. Taken with our data, these observations suggest that the present nascent cardiomyocytes acquired tetrodotoxin-sensitivity during the culture, similar to the *in vivo* development of cardiomyocytes. In agreement, our time-course morphological studies show functional localisation and re-localisation of several cardiac proteins.

Clusters of nascent cardiomyocyte responded to isoprenaline, acetylcholine and intrinsic bioactive peptides in a manner similar to what physiological isolated cardiomyocytes do *in vitro*^26,27^. It indicates that these major plasma membrane receptors are also expressed properly and that their downstream intracellular signalling pathways worked properly in the nascent cardiomyocyte clusters.

We determined whether fibroblasts are recruited to generate cardiomyocytes during SANC culture because our time-lapse video images showed gathering of flat and thin fibroblast-like cells and their growth on the rims of beating clusters. To this end, we purified and labelled GCFs and then co-cultured these with SANCs. Under these conditions, GCFs that reached in close proximity to SANCs started to beat spontaneously and expressed cardiac proteins, including cTnT and desmin. Moreover, the expression of cardio-specific homeoprotein Nkx2.5^28,29^ was demonstrated in GCFs from 48-h co-culture with SANCs. Because Nkx2.5 is expressed in cardiomyocytes from early developmental stages and throughout adulthood^28–30^, these results indicate that SANCs generated cardiomyocytes by recruiting and trans-differentiating cardiac fibroblasts at the rims of clusters, thereby gradually expanding the beating clusters. However, even if the culture was continued, GCFs, in which Nkx2.5 expression was detected, never started to beat. This is probably because the expressed Nkx2.5 was lost by binding to the reporter gene for Nkx2.5, so that Nkx2.5 could not keep functioning as a transcription factor any more. Despite the reporter gene indicated the early stage of cardiomyocyte differentiation, it may, in turn, possibly have acted as a functional absorber of Nkx2.5. This awaits further study. Moreover, expression of Nkx2.5 is merely a start point for cardiac differentiation, after which time a whole set of cardiac proteins would be gradually expressed to enable the cells to beat.

Cardiac progenitor cells, including cardiac side population cells and other cardiac stem cells, have the potential to differentiate into cardiomyocytes after treatments with oxytocin^8^ and 5-azacytidine or trichostatin A^8,31^, respectively. In the present study, oxytocin and trichostatin A did not augment cTnT expression significantly in SANC cultures (Supplementary Fig. S5) nor did these agents promote the expression of cardiac proteins, such as desmin or cTnT, in purified GCFs in monocultures. Hence, if present in isolated SANC suspensions, cardiac stem cells are unlikely to differentiate into the cardiomyocytes of beating clusters. Proliferation of originally placed nodal cells is also unlikely as a mechanism for the construction of the present beating clusters because SANCs and atrial myocytes are highly differentiated. Our time-laps video recordings showed no such features of proliferating cells. We also contend that our expanding beating cardiomyocyte clusters are not solely the product of fusions of SANCs and GCFs, although further studies will clarify the contributions of other processes. Taken together, our observations and considerations suggest that the generation of beatable cardiomyocyte clusters by SANCs does not follow the differentiation of cardiac progenitor cells or stem cells or reflect the proliferation or aggregation of cardiomyocytes that exist differentiated in cultures.

To decipher these processes further, we co-cultured SANCs and GCFs in chambers that were separated by cellulose membranes. Under these non-contact conditions, GCFs expressed the cardiac cytoskeleton protein desmin^32^, but did not express cTnT. Moreover, extracellular electrical field stimulation caused cTnT expression in GCFs only in the presence of culture medium that was pre-conditioned by SANCs. In contrast, GCFs failed to induce cTnT expression in cultures with formaldehyde-fixed SANCs, even in the presence of SANC-conditioned medium. Here, results with using other combinations of conditioned medium, dead SANCs and electrical stimulation, which failed to express cTnT in labelled GCFs, are also provided in Supplementary Fig. S6. Experimentally, panel a in this figure is regarded to be a negative control for cTnT expression in GCFs, cultured without SANCs. Finally, spontaneous beating of GCFs was not established in the absence of cell–cell contacts with living SANCs. Thus, although humoral factor(s) and electrical signals from SANCs are found to be necessary to induce the expression of desmin and cTnT, these conditions may not be sufficient to establish contractions in these nascent cardiomyocytes.

Previous studies have shown that stem cells acquire cardiac phenotypes and express cardiac-specific genes, but only when in direct contact with adjacent myocytes^33,34^. Accordingly, in heart tissues, action potentials are propagated through direct cell-to-cell contacts with adjacent cardiac cells. We demonstrated oscillations of intracellular Ca^2+^ and direct cell-to-cell communication between the cardiomyocytes of our clusters. In addition, pharmacological inhibition of Na^+^ channel activation, blockade of Ca^2+^ entry and inhibition of intracellular Ca^2+^ release suppressed cluster formation. Hence, direct cell-to-cell communication between SANCs with GCFs is essential for cardiac differentiation of GCFs and their Ca^2+^ oscillations, which are likely initiated by electrical excitation of SANCs.

Pharmacological inhibition of intracellular signal pathways suggested that activation of ROCK, PI_3_K and GSK3 promotes cardiomyocyte differentiation in SANC cultures and that activation of ERK1/2, p38 and PKA suppresses this process. Although Ca^2+^ oscillations were also essential for the recruitment of cardiomyocytes, inhibition of CaMK II did not affect cTnT expression during cluster formation. Whereas EGF and VEGF enhanced cardiomyocyte generation by activating Akt in gene transfer experiments^20^, inhibition of Akt did not reduce cTnT expression. A limitation of the present study is that pharmacological experiments do not distinguish target cells of inhibitor agents. Our results do, however, indicate that ROCK, PI_3_K and GSK3 operate in essential intracellular signal pathways to upregulate cardiac gene transcription in GCFs.

In summary, we found that SANCs from adult guinea pig hearts recruit fibroblasts and promote their differentiation into cardiomyocytes in long-term primary cultures. We also show that trans-differentiation of fibroblasts into cardiomyocytes is mediated by humoral factor(s), electrophysiological activities, and direct cell-to-cell communications. Because cardiac pacemaker cells are present in limited numbers in adult hearts^12^ and sinoatrial nodes need efficient cell turnover during the lifetime of the body, the potential of SANCs to generate cardiomyocytes in their vicinity may play a role in maintaining pacemaker cells in sinoatrial nodes *in vivo*. This finding may also provide a key to supplying cardiomyocytes for cardiac regenerative therapies in addition to reprogramming of fibroblast cells through direct gene transfer and using chemical agents^13,14,16^.

## Methods

All animal procedures were approved by the Teikyo University Chancellor’s Animal Research Committee (Permit Number 12-031). We confirm that all methods described below were performed in accordance with the relevant guidelines and regulations.

### Cell isolation and culture

SANCs were enzymatically isolated from adult male guinea pigs (*Cavia porcellus*, 6–8 weeks old, 450–500 g body weight, unless otherwise indicated, Sankyo Labo Service Inc. Japan) using collagenase and elastase as previously described, with slight modifications^35^. Isolated SANCs were then re-suspended in high-glucose Dulbecco’s Modified Eagle Medium (0.45% glucose) supplemented with 10% FBS (MP Biomedicals, Inc.), and 2-mL suspensions were then placed in 35-mm culture dishes. Left atrial cells of adult guinea pigs were isolated using collagenase as described previously^27^ and were cultured in the same way. GCFs were isolated from the left atrium using collagenase, purified as described previously^36^ and used after more than 5 passages to avoid contamination of cardiomyocytes. We define cells obtained by this method, which were vimentin-positive, as GCF in the present study. Isolated SANCs, left atrial cells and GCFs were cultured at 37 °C in an incubator aerated with 5% CO_2_. Culture media were replaced every 3 days.

### Measurements of beating cell areas

Microscope images of spontaneously beating cell clusters were obtained using an inverted phase-contrast microscope (Nikon) and were recorded on a digital video cassette recorder (VCR). Still photo images were then picked from video data and were stored on a Macintosh computer. Outer rims of contracting clusters were traced from still images of clusters on the computer screen with reference to the videos, and areas of spontaneously beating cell clusters were quantified using ImageJ software.

### Quantification of beating rates of cell clusters

Light phase-contrast microscope video images of spontaneously beating cell clusters were recorded on a digital VCR. Movements of beating clusters were converted to analogue waveform signals using a video dimension analyser (VDA; model 303, Instrumentation for Physiology and Medicine) and were recorded digitally on PowerLab (AD Instruments) to count beat rates.

### Time-Lapse morphological observations of SANCs in culture

To record morphological changes in SANCs over culture times, cell images were automatically captured and recorded in 30 min intervals from 2 to 284 h after the start of culture using a BioStation IM microscope/incubator (Nikon) at 37 °C and in the presence of 5% CO_2_.

### RNA Extraction and RT-PCR

Total RNA was extracted from cell clusters that were grown in SANC cultures for 3 weeks and was reverse transcribed using standard methods. Subsequently, PCR analyses were performed using the resulting cDNA and specific primers (Supplementary Table S1) with 25–35 cycles. Glyceraldehyde-3-phosphate-dehydrogenase (*GAPDH*) or *cTnT* were used as internal controls.

Expression levels were estimated using standard quantitative RT-PCR with an Applied Biosystems 7500 Real-Time PCR System. In experiments using quantitative RT-PCR, estimated mRNA expression values were expressed as percentages of corresponding mRNA expression levels in internal control dishes for each experiment.

### Immunocytochemistry

Cultured cells were fixed with 2% paraformaldehyde in 100-mM phosphate buffer solution for 30 min at 4 °C. After permeabilisation with 0.1% triton X-100, primary antibodies were applied overnight at 4 °C in phosphate buffer. After washing out primary antibodies, Alexa 488- or 546- conjugated secondary antibodies were applied for 60 min at room temperature (Supplementary Table S2). We confirmed by fundamental experiments that there is no non-specific binding of the secondary antibodies to the materials. Immunocytochemically stained cell images were recorded using an inverted confocal laser scanning microscope (Leica CLSM TCS SP5).

### Electrophysiological recording of membrane potential

Membrane potentials were recorded from cell clusters using patch-clamp techniques in the whole-cell configuration. Experiments were performed with an EPC-7 (List Electronic) patch-clamp amplifier and pCLAMP 7 software (AXON Inc.) as described elsewhere^26,27,35^.

### Ca^2+^ imaging

Cell clusters that emerged during SANC culture were incubated in normal Tyrode solution containing 5-mg/mL Fluo 4-AM, 0.04% pluronic F127 and 1-mM probenecid as a loading solution at 37 °C in the dark for 1 h. After washing out the loading solution with normal Tyrode solution containing 1-mM probenecid, cell clusters were placed on the stage of an inverted fluorescent microscope (Nikon) and excited at 450–490 nm using a dichroic mirror. Images of emissions at > 520 nm were recorded using a digital VCR for later analysis.

### Pre-labelling of GCFs with EGFP

GCF cell lines that stably express EGFP were prepared by transfecting GCFs with an EGFP gene and a neomycin resistance gene. The stably transfected clones were selected using 300 µg/mL G418 (Calbiochem).

### Nkx2.5 Reporter and GCFs

The pEGFP/Nkx2.5BD reporter system was constructed by sub-cloning 3 tandem copies of the Nkx2.5 binding sequence (5′-CCCGGGAGTTAATTGCGTAGTTAATTGCAGCAGTTAATTGCAGATCT-3′)^37^ into a pEGFP-TK vector containing the thymidine kinase (TK) minimal promoter linked to *Enhanced Green Fluorescent Protein* (*EGFP*) (Supplementary Fig. S11). Purified GCFs were then transfected with pEGFP/Nkx2.5BD or an empty vector using Lipofectamine^TM^ 2000 reagent.

### Roles of intracellular signals

The roles of intracellular signalling cascades in cardiomyocyte generation were examined by exposing SANCs to various selective chemical inhibitors or stimulants for 3 weeks in culture (Supplementary Table S3).

### Western blotting

Total protein was extracted from cell clusters after 3 weeks culture. The protein extracts (50 µg) were then fractionated using sodium dodecyl sulphate-polyacrylamide gel electrophoresis (SDS-PAGE) with 10–20% gradient gels. Electrophoresed proteins were transferred to polyvinylidine difluoride (PVDF) membranes, and primary antibodies (Supplementary Table S2) were applied overnight at 4 °C. After washing in PBS, alkaline phosphatase-conjugated secondary antibody (Vector) was applied for 1 h at room temperature, and proteins were detected using standard staining procedures.

### Induction of cardiac differentiation in GCFs

GCFs were purified and co-cultured with isolated SANCs for 3 weeks in the same culture dishes but were physically separated by cellulose membranes with 1-µm pores. To investigate the role of physical contact between SANC membranes and GCFs, GCFs were cultured with SANCs that had been pre-fixed with 2% paraformaldehyde for 30 min at 4 °C. Continuous electrical field stimulation was applied to GCFs during culture with consecutive rectangular pulses (30 V) of 5 ms at 1 Hz using a pair of platinum electrodes with an electronic stimulator and an isolator. The efficiency of field stimulation was confirmed by contraction of isolated ventricular cardiomyocytes (Supplementary Movie S8).

### Statistics

All values are expressed as mean ± standard errors of the mean (S.E.M). Differences were identified using analysis of variance (ANOVA) followed by Dunnett’s or Tukey’s tests. Time-dependent changes in beating areas were statistically compared using repeated measures ANOVA with JMP software version 8 (SAS Institute Inc.). Differences were considered significant when *P* < 0.05.

## Supplementary information

Supplementary Movie S1

Supplementary Movie S2

Supplementary Movie S3

Supplementary Movie S4

Supplementary Movie S5

Supplementary Movie S6

Supplementary Movie S7

Supplementary Movie S8

Dataset 1

## Data Availability

All data generated or analysed during this study are included in this published article (and its Supplementary Information files). The datasets generated during and/or analysed during the current study are available from the corresponding author on reasonable request.
