# Supplementary material for: Cardiac Pacemaker Cells Generate Cardiomyocytes from Fibroblasts in Long-Term Cultures
Source: Sci Rep. 2019 Oct 23;9:15174. doi: 10.1038/s41598-019-51001-6 (PMC6811548; doi:10.1038/s41598-019-51001-6)
Supplement: Supplementary file 9 — Dataset 1 [file 41598_2019_51001_MOESM9_ESM.pdf]

Supplementary Information

Cardiac Pacemaker Cells Generate Cardiomyocytes from Fibroblasts in Long-Term Cultures

Shigeki Kiuchi, Akino Usami, Tae Shimoyama, Fuminori Otsuka, Sachiko Yamaguchi, Tomonori Nakamura, Shigeto Suzuki & Kageyoshi Ono

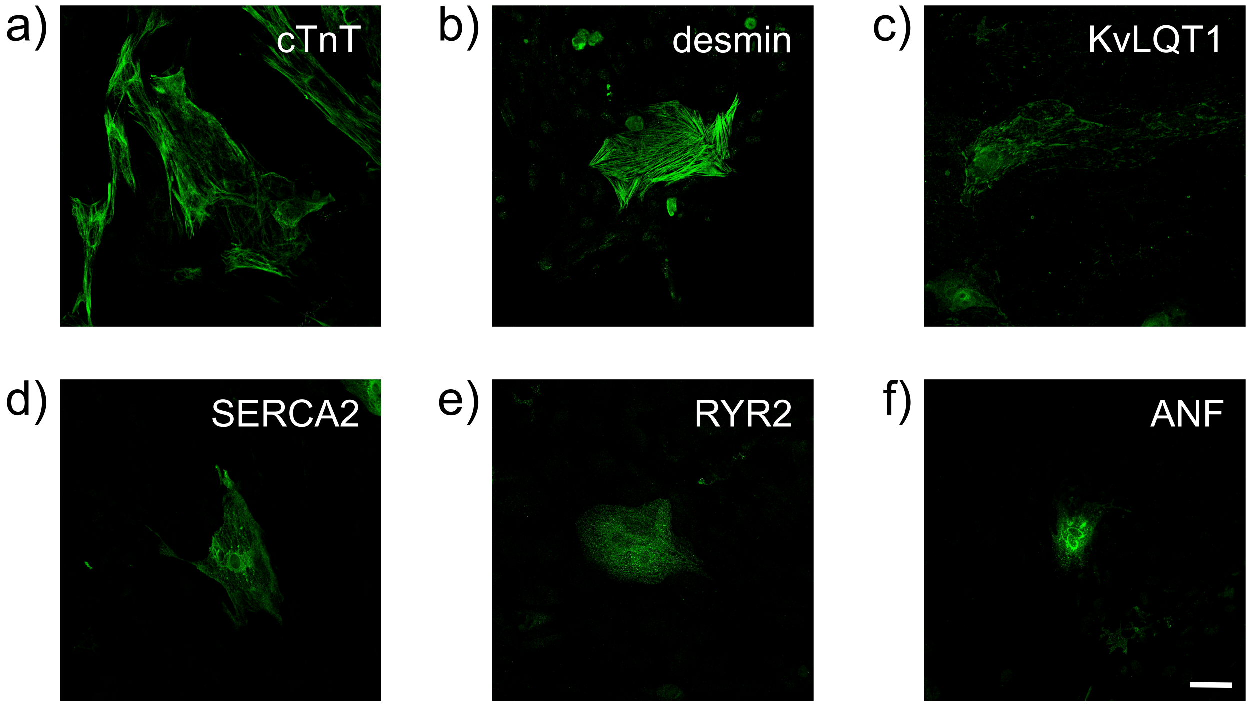

Figure S1. Immunocytochemical detection of cardiac proteins in nascent cell clusters. The cell clusters, which had grown around the SANCs after 1 week of culture, expressed cTnT (a), desmin (b), KvLQT1 (c), SERCA2 (d), RYR2 (e), and ANF (f). Fiber-like construction was observed for cTnT (a) and desmin (b). *Bar*, 50 µm.

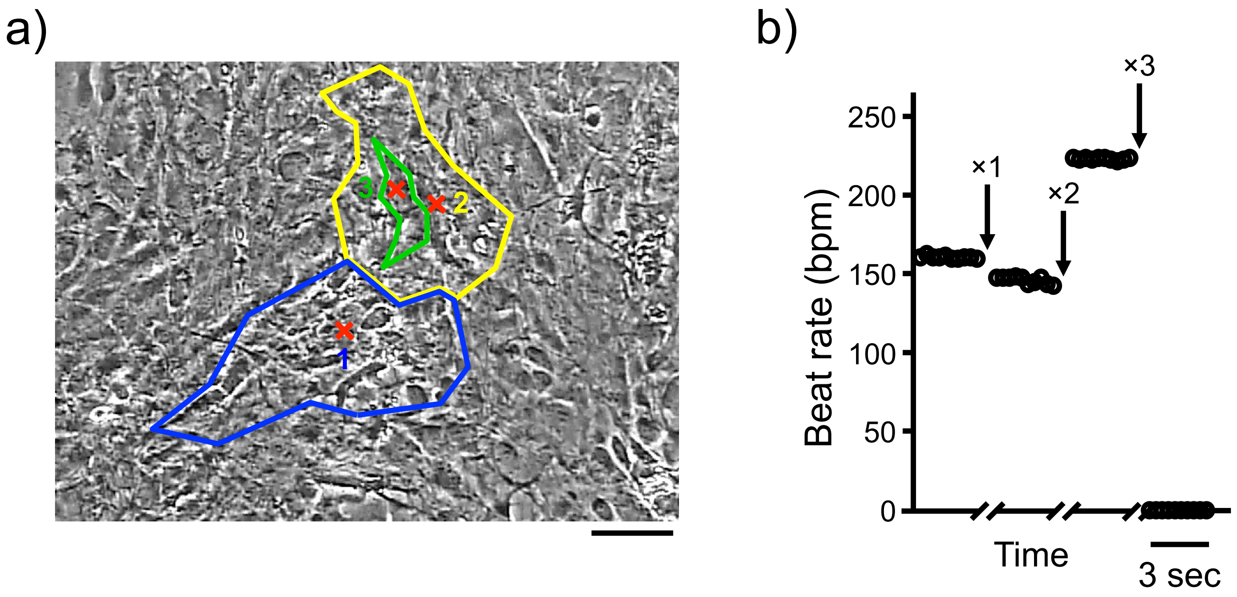

Figure S2. Presence of both spontaneously beating and passively driven cells in a nascent cardiomyocyte cluster. Typical phase-contrast image cropped from video recording of a beating cluster of cardiomyocytes (a) and the overall rate of its spontaneous beating (b). This beating cluster was observed after 3 weeks in culture of SANCs. Consecutive crashing of three central cells, denoted by ×1, ×2 and ×3, stopped the beating part by part, as marked by polygons blue, yellow and green in a raw, and changed the beat rate as crashing progressed, finally ceasing the beating of the whole cluster. Thus, three central pacemaking cells that drove surrounding quiescent cardiomyocytes were found to exist in each of three synchronized beating clusters. *Bar*, 50 µm.

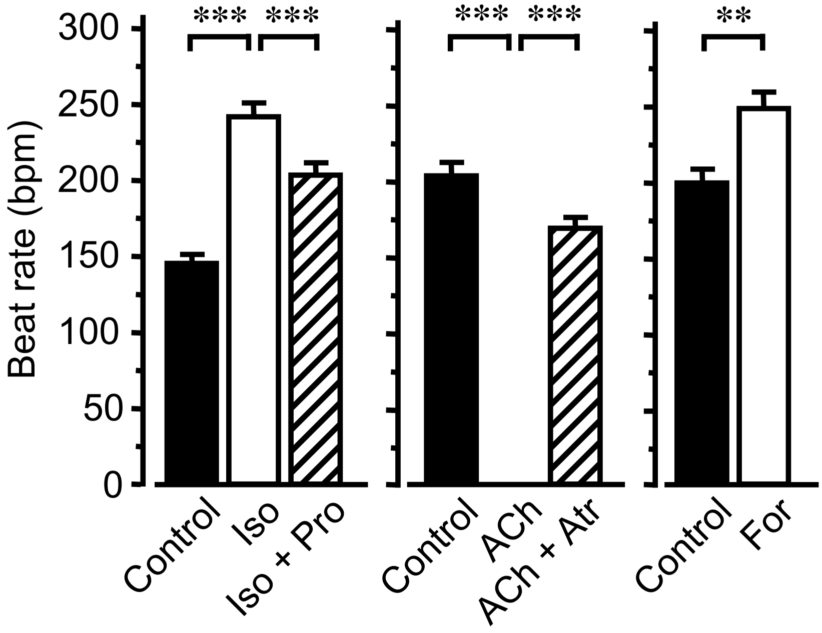

Figure S3. Pharmacological characterisation of the nascent cardiomyocyte clusters – (1). Positive and negative chronotropic responses of nascent cardiomyocyte clusters to isoprenaline (Iso, 0.1 μM, left panel) and acetylcholine (ACh, 1 μM, middle panel) and their blockade by the antagonists propranolol (Pro, 0.3 μM) and atropine (Atr, 0.1 μM), respectively; right panel, increase in beat rates by forskolin (For, 3 μM). Data are summarised from 22 independent experiments and expressed as mean ± S.E.M. Vertical bars denote S.E.M.; Tukey's test; $^{**}P < 0.01$, $^{***}P < 0.001$.

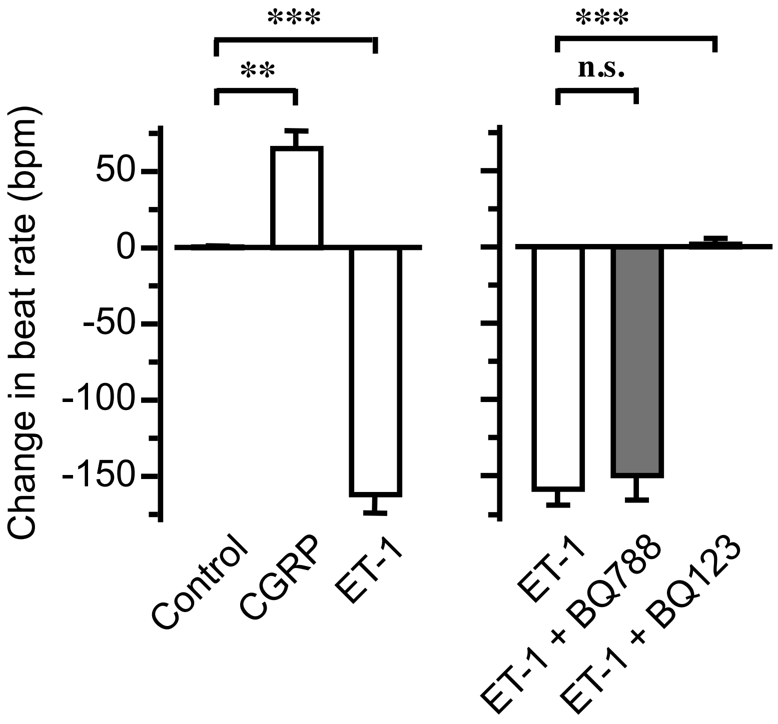

Figure. S4. Pharmacological characterisation of the nascent cardiomyocyte clusters – (2). Changes in beating rate in response to CGRP (0.1 μM, left panel) and endothelin-1 (0.1 μM, both panels) are illustrated. The negative chronotropic response to endothelin-1 was completely blocked by an ET_A receptor antagonist BQ123 (1 μM, n = 3); however, it was not blocked by an ET_B receptor antagonist BQ788 (3 μM, n = 3). Data are expressed as mean ± S.E.M. Vertical bars denote S.E.M.; Dunnett's test; n.s., not significant; $**P < 0.01$, $***P < 0.001$, compared with the control.

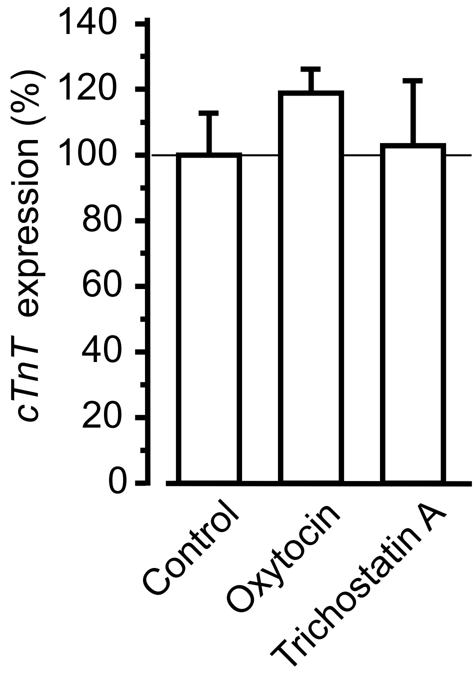

Figure S5. Changes in *cTnT* expression levels during SANC culture by differentiation-promoting substances. Effect of oxytocin (100 nM) or trichostatin A (10 pg/mL) (n = 3) on *cTnT* expression is illustrated. Neither oxytocin nor trichostatin A increased expression of *cTnT* significantly. 5-azacytidine was toxic so that the corresponding data was not obtained. The mRNA levels were measured by quantitative RT-PCR and the values are expressed as per-cent of each mRNA level measured in the internal control dish in each experiment.

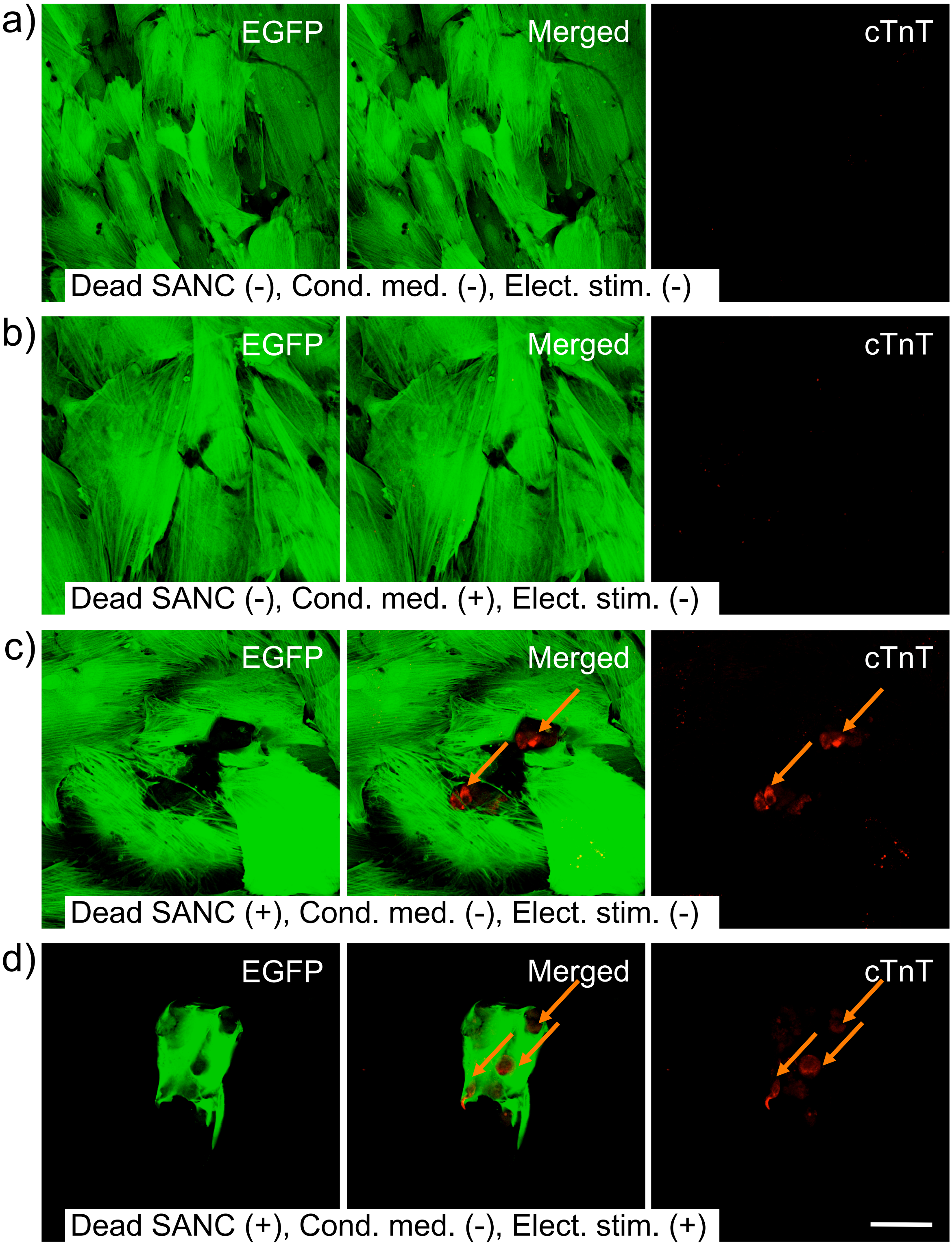

Figure S6. Differentiation of GCFs placed in a simulated cardiac microenvironment. Immunocytochemical detection of cTnT protein (red) in EGFP-labelled (green) GCFs cultured under various conditions for 1 week; a) GCFs cultured in untreated conditions; b) GCFs cultured in SANC-conditioned medium; c) co-culture with pre-fixed (dead) SANC (arrow); d) co-culture with pre-fixed SANC (arrow) under extracellular electric field stimulation; cTnT was detected only in pre-fixed SANCs (arrows). *bar*, 50 μm.

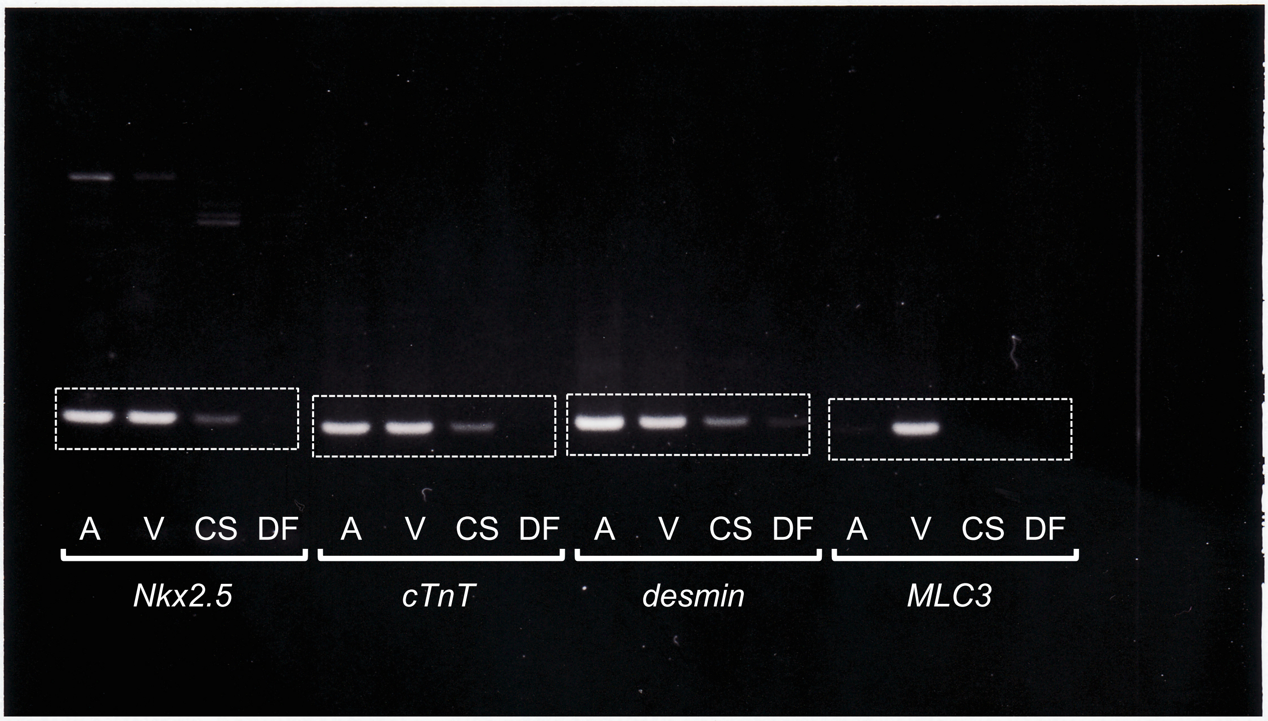

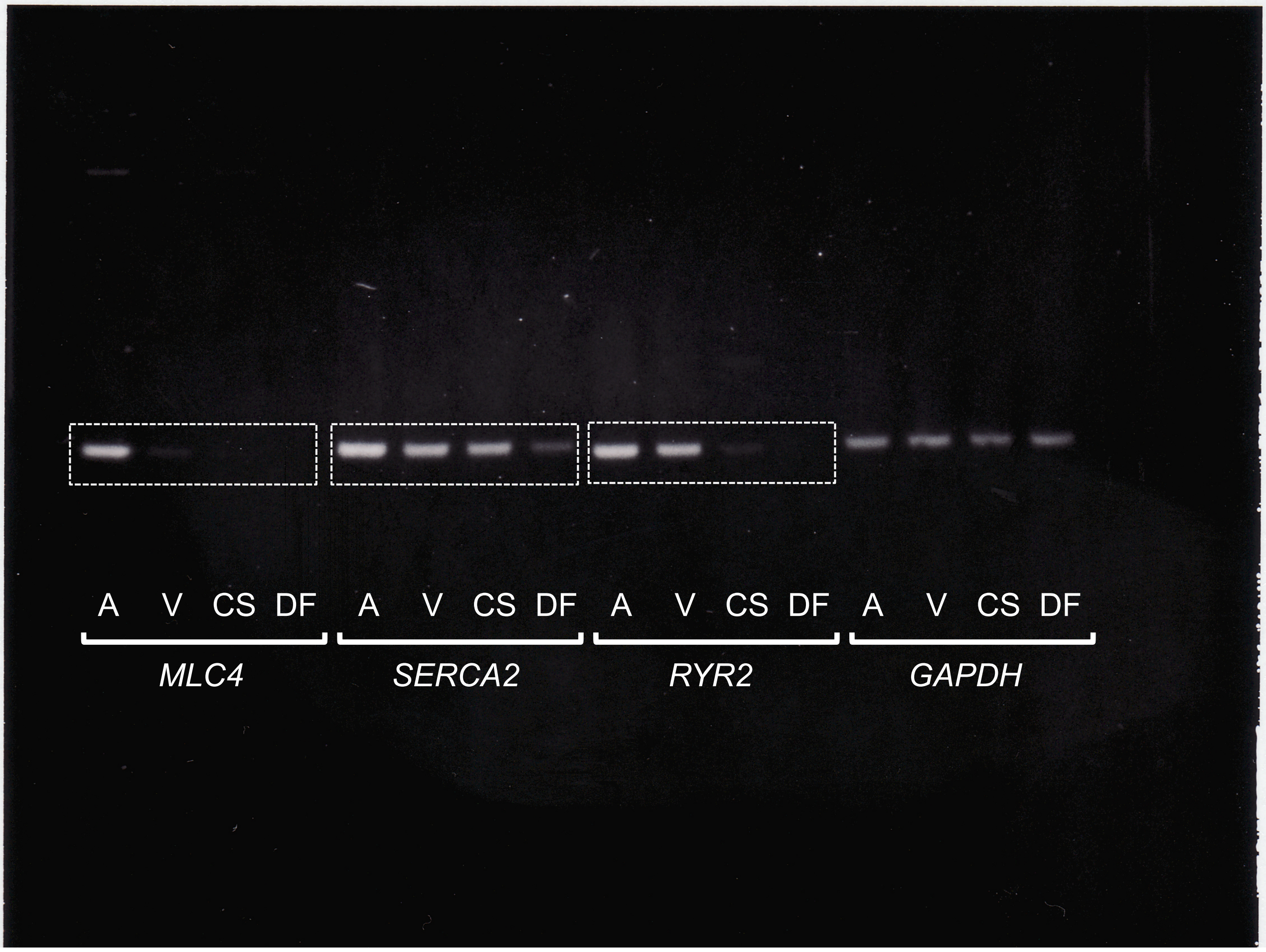

Figure S7. Whole gel electrophoresis patterns of RT-PCR products illustrated in Figure 2A-a). White dotted squares show the cropped areas on the gels used for Figure 2A-a), where the brightness was slightly raised equally for all the crops for clarity. The contrast was not changed during the processing of these bands. Each band was retrieved to determine its base sequence and matched anticipated base length described in Supplementary Table S1. See also Figure S8 for MLC3, MLC4 and HCN4.

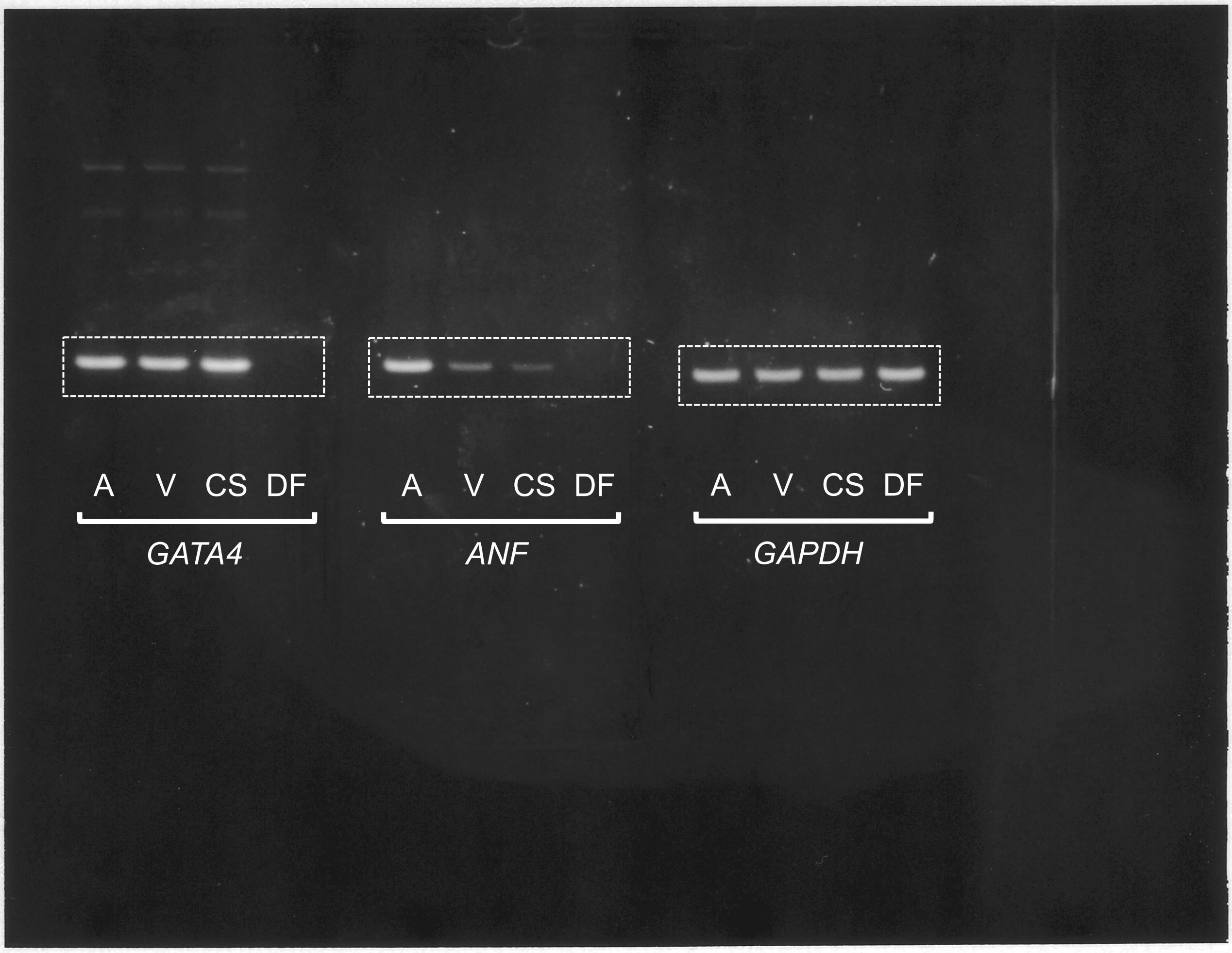

Figure S8. Whole gel electrophoresis patterns of RT-PCR products illustrated in Figure 2A-a). White dotted squares show the cropped areas on the gels used for Figure 2A-a), where the brightness was slightly raised equally for all the crops for clarity. The contrast was not changed during the processing of these bands. Each band was retrieved to determine its base sequence and matched anticipated base length described in Supplementary Table S1.

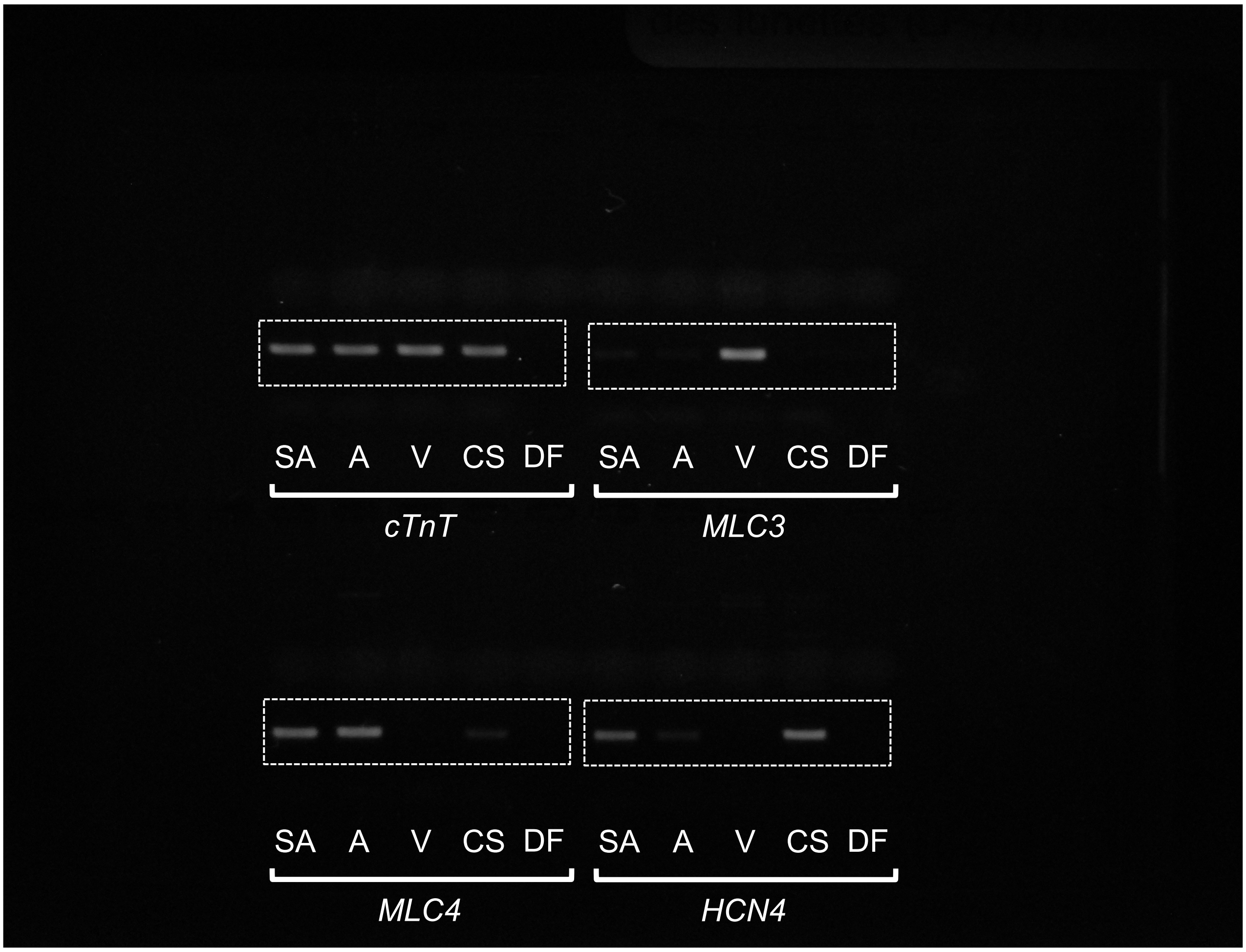

Figure S9. Whole gel electrophoresis images of RT-PCR products illustrated in Figure 2A-b). White dotted squares show the areas on the gels cropped for Figure 2A-b), where the brightness of the whole cropped areas was slightly increased equally for all of the 4 crops for clarity. Contrast was not changed during the processing of these bands.

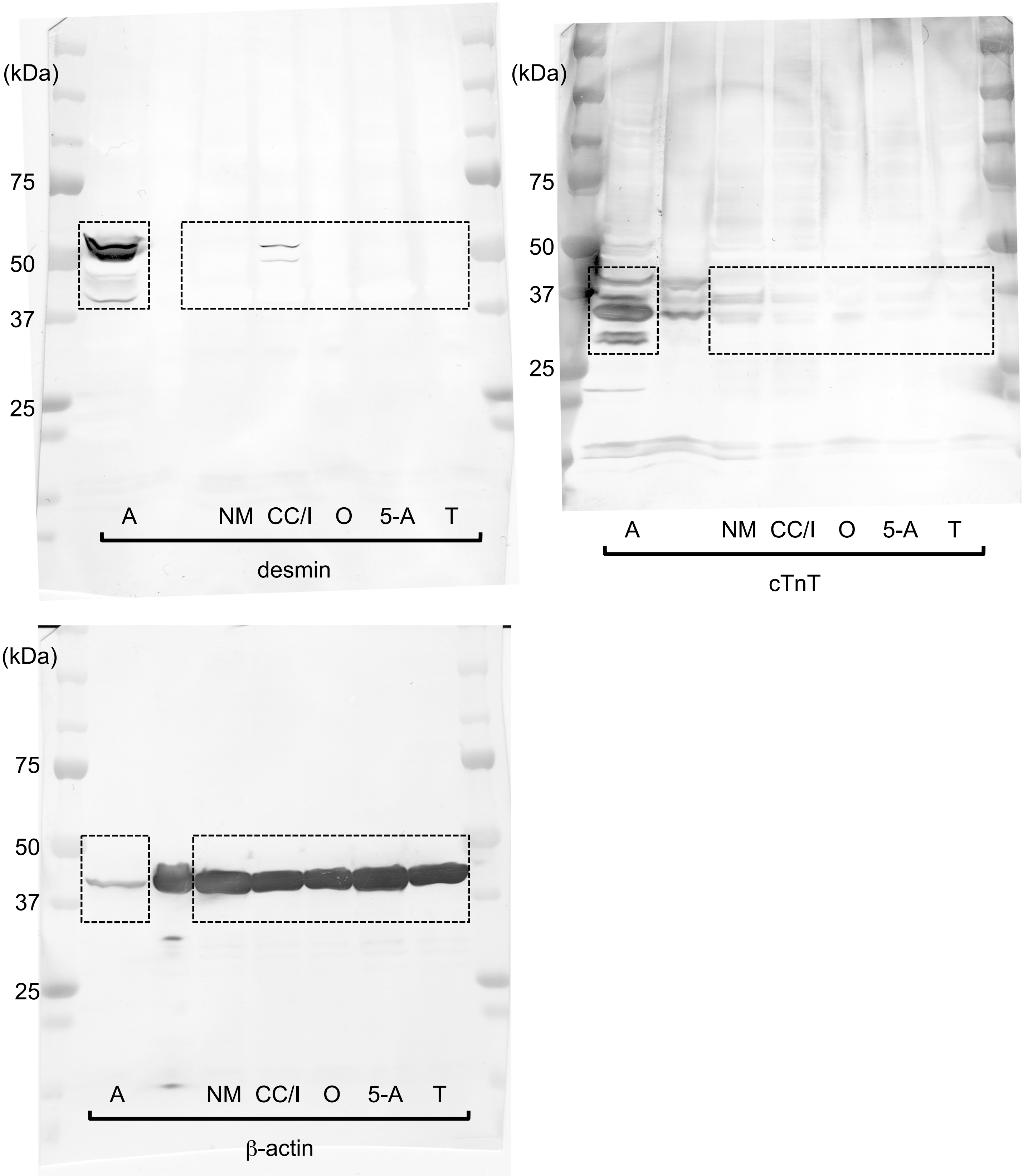

Figure S10. Full length blots of Figure 7A. Dotted squares indicate the areas cropped for Figure 7A. No adjustment in either brightness or contrast was made during the processing of these images of blots to construct Figure 7A.

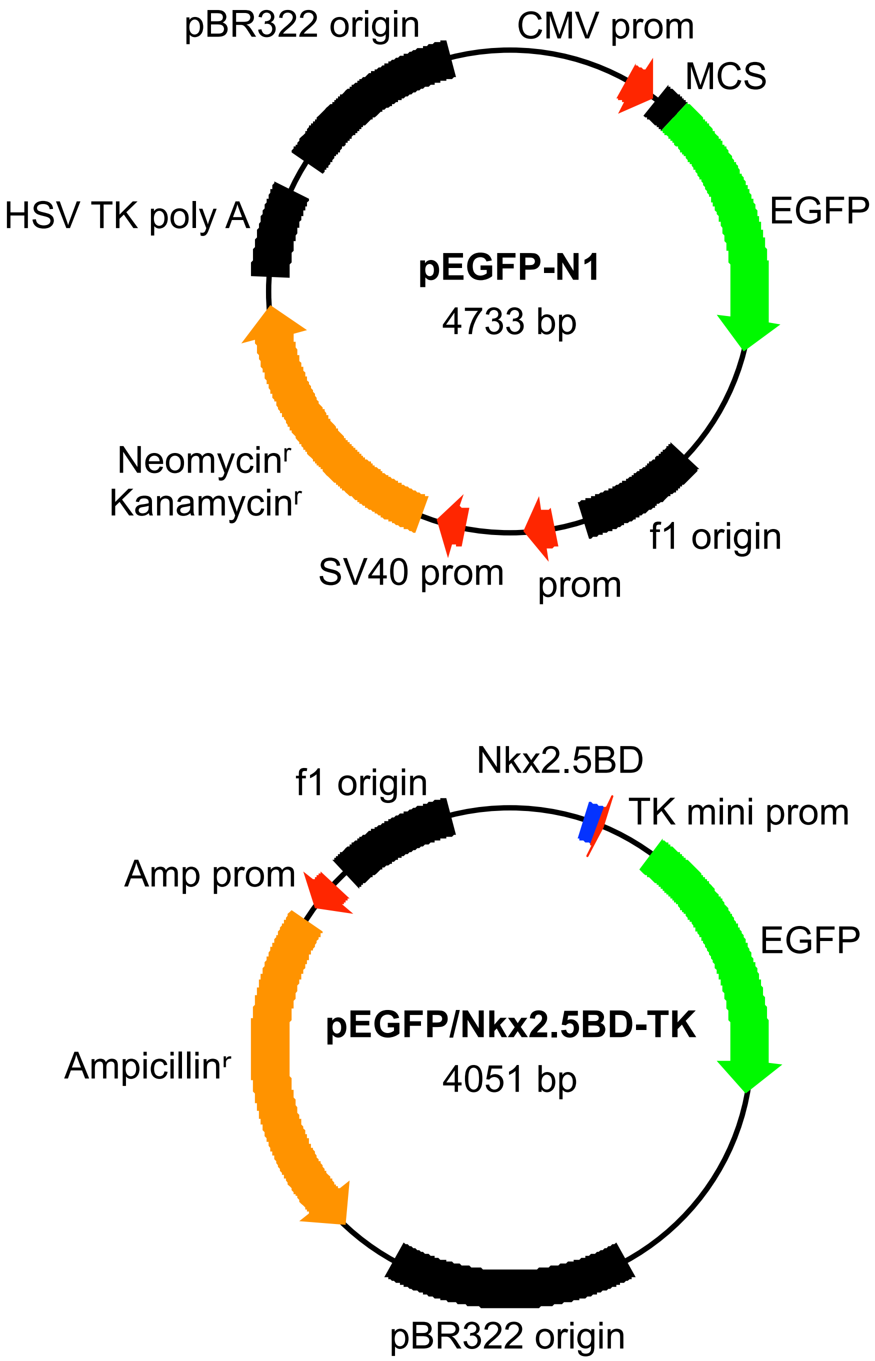

Figure S11. Plasmids used in the present study. pEGFP-N1 was used for pre-labelling of GCF with EGFP. pEGFP/Nkx2.5BD reporter system was constructed by sub-cloning 3 tandem copies of the Nkx2.5 binding sequence into a pEGFP-TK vector containing the thymidine kinase (TK) minimal promoter linked to *EGFP*.

Target gene	Sequence (5'-3') Forward and Reverse primers	Annealing temperature	Product size
Nkx2.5	CTAAGGACCCCAGAGCGGATAAG<br>GACAGGTACCGCTGTTGCTTG	60 °C	184 bp
GATA4	AGGGGATTCAGACCAGAAAACG<br>CTGCCATGCCCATAGTGAGAG	60 °C	199 bp
cTnT	GCAGACAGAGAGGGAAAAGAAAAAG<br>CCGGAGAACATTGATTTCGTATTTC	60 °C	193 bp
desmin	ATCTCTCAACGAGGAAATTGCATTC<br>GTTCTTAGCTGCGATGGTCTCATAC	60 °C	175 bp
MLC3	CAGCACATCTCCAAGAACAAGG<br>TTCTCCACCTCGTCTTCAGTCAG	60 °C	158 bp
MLC4	AGTTCAAGGAGGCCTTTTCATTG<br>GAAGTCCAGCATCTTGGTGTTC	60 °C	176 bp
HCN4	CCTCATCCAGGTCTCCATCATC<br>AAAGGCTACAGGGGAAGCCACC	68 °C	170 bp
SERCA2	CATGGGAGAAGTGCATAAAGACG<br>CTTCTCAACCAGGCAAGTGAGAG	60 °C	181 bp
RYR2	TCCGTTCTCTGCTGAGTGTAAGG<br>ACTCTCCACCTCCAAGGACATTC	60 °C	174 bp
ANF	GATGCCGTTAGAAGACGAGGTG<br>CGCTCTCAGCTTGCTTTTCAG	60 °C	202 bp
GAPDH	ATACGATGACATCAAGAAGGTGGTG<br>ATACCAAGAAACGAGCTTGACAAAG	60 °C	181 bp

Table S1. Detection primers for target genes and the PCR conditions used in the present study.

a)

Antibody	Company	Conc.
Mouse anti-Troponin T monoclonal antibody	Thermo	1 : 100
Mouse anti-Desmin monoclonal antibody	IMMNOTECH	1 : 10
Rabbit anti-KvLQT1 polyclonal antibody	CHEMICON	1 : 500
Mouse anti-SERCA2 ATPase monoclonal antibody	Affinity BioReagents	1 : 500
Mouse anti-Ryanodine receptor monoclonal antibody	Affinity BioReagents	1 : 200
Rabbit anti-Atrial natriuretic peptide monoclonal antibody	CHEMICON	1 : 100
Alexa Flour 488 goat anti-mouse IgG	Invitrogen	1 : 500
Alexa Flour 488 goat anti-rabbit IgG	Invitrogen	1 : 500
Alexa Flour 546 goat anti-mouse IgG	Invitrogen	1 : 500

b)

Antibody	Company	Conc.
Mouse anti-Desmin monoclonal antibody	IMMNOTECH	1 : 50
Mouse anti-Troponin T monoclonal antibody	Thermo	1 : 500
Mouse anti-baction monoclonal antibody	Applied Biological Materials	1 : 1000
Alkaline phosphotase anti mouse IgG	VECTOR	1 : 1000

Table S2. Primary antibodies and secondary antibodies used in the immunocytochemistry (a) and western blot analyses (b).

a)

Reagent	Target	Conc.	Use
Ivabradine	I_f channel	0.1 μM, 1 μM	Fig. 3
CsCl	I_f channel	3 mM	Fig. 3
E-4031	I_{Kr} channel	3 μM	Fig. 3
KB-R7943	$I_{Na/Ca}$ exchanger	5 μM	Fig. 3
Ni^{2+}	I_{CaT} channel	50 μM	Fig. 3
Nicardipine	I_{CaL} channel	10 μM	Fig. 3, 5
Tetrodotoxin	I_{Na} channel	10 μM	Fig. 3, 5
2-APB	Inositol 1,4,5-trisphosphate receptor	3 μM	Fig. 5
H89	Protein kinase A	10 μM	Fig. 6
GF109203	Protein kinase C	1 μM	Fig. 6
KT5823	Protein kinase G	400 nM	Fig. 6
KN-93	Calcium/calmodulin-dependent protein kinase II	2 μM	Fig. 6
Y-27632	Rho-associated kinase	10 μM	Fig. 6
AG1478	Epidermal growth factor receptor kinase	1 μM	Fig. 6
LY294002	Phosphatidylinositol 3-kinase	10 μM	Fig. 6
Akt inhibitor VIII	Akt (protein kinase B)	1 μM	Fig. 6
BIO	Glycogen synthase kinase 3	3 μM	Fig. 6
PD98059	Extracellular signal regulated kinase 1/2	10 μM	Fig. 6
SP600125	c-Jun N-terminal kinase	10 μM	Fig. 6
SB203580	p38 MAP kinase	10 μM	Fig. 6
5-azacytidine	DNA methyltransferase	10 μM	Fig. 7
Trichostatin A	Histone deacetylase	10 pg/mL	Fig. 7
Propranolol	Adrenergic β receptor	0.3 μM	Fig. S3
Atropine	Muscarinic M receptor	0.1 μM	Fig. S3
BQ123	Endothelin A receptor	1 μM	Fig. S4
BQ788	Endothelin B receptor	3 μM	Fig. S4

b)

Reagent	Target	Conc.	Use
8-bromo cAMP	Protein kinase A	500 μM	Fig. 6
Phenylephrine	Protein kinase C	3 μM	Fig. 6
8-bromo cGMP	Protein kinase G	500 μM	Fig. 6
BMP4	Bone morphogenetic protein 4	10 ng/mL	Fig. 6
IGF	Insulin-like growth factor	10 ng/mL	Fig. 6
EGF	Epidermal growth factor	100 ng/mL	Fig. 6
VEGF	Vascular endothelial growth factor	50 ng/mL	Fig. 6
Triiodothyronine	Triiodothyronine receptor	30 nM	Fig. 6
Retinoic acid	Retinoic acid receptor	1 μM	Fig. 6
Oxytocin	Oxytocin receptor	100 nM	Fig. 7
Isoprenaline	Adrenergic β receptor	0.1 μM	Fig. S3
Acetylcholine	Muscarinic M receptor	1 μM	Fig. S3
Forskolin	Adenylyl cyclase	3 μM	Fig. S3
CGRP	Calcitonin gene-related peptide receptor	0.1 μM	Fig. S4
Endothelin-1	Endothelin receptor	0.1 μM	Fig. S4

Table S3. Inhibitors (a) and stimulants (b) used in the present study.
